# Compound stress response in stomatal closure: a mathematical model of ABA and ethylene interaction in guard cells

**DOI:** 10.1186/1752-0509-6-146

**Published:** 2012-11-25

**Authors:** Mariano Beguerisse-Dıaz, Mercedes C Hernández-Gómez, Alessandro M Lizzul, Mauricio Barahona, Radhika Desikan

**Affiliations:** 1 Department of Life Sciences, Imperial College London, London, SW7 2AZ, UK; 2 Department of Mathematics, Imperial College London, London, SW7 2AZ, UK

**Keywords:** ROS, Guard cells, Stomatal closure, Ethylene, ABA, Combined stimuli, Antioxidants, Mathematical modelling, ODE models

## Abstract

**Background:**

Stomata are tiny pores in plant leaves that regulate gas and water exchange between the plant and its environment. Abscisic acid and ethylene are two well-known elicitors of stomatal closure when acting independently. However, when stomata are presented with a combination of both signals, they fail to close.

**Results:**

Toshed light on this unexplained behaviour, we have collected time course measurements of stomatal aperture and hydrogen peroxide production in *Arabidopsis thaliana* guard cells treated with abscisic acid, ethylene, and a combination of both. Our experiments show that stomatal closure is linked to sustained high levels of hydrogen peroxide in guard cells. When treated with a combined dose of abscisic acid and ethylene, guard cells exhibit increased antioxidant activity that reduces hydrogen peroxide levels and precludes closure. We construct a simplified model of stomatal closure derived from known biochemical pathways that captures the experimentally observed behaviour.

**Conclusions:**

Our experiments and modelling results suggest a distinct role for two antioxidant mechanisms during stomatal closure: a slower, delayed response activated by a single stimulus (abscisic acid ‘or’ ethylene) and another more rapid ‘and’ mechanism that is only activated when both stimuli are present. Our model indicates that the presence of this rapid ‘and’ mechanism in the antioxidant response is key to explain the lack of closure under a combined stimulus.

## Background

**Figure 1:**
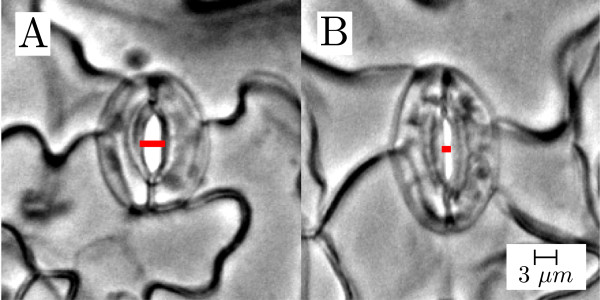
**Stomata from the model organism *Arabidopsis thaliana *.** Stomata may be in an open **(A)** or closed **(B)** state, as required by the plant. The lines across the pore opening indicate the measure of stomatal aperture used in our experiments.

Stomata are tiny pores located mainly in the lower epidermis of plant leaves. Each stoma is formed by two guard cells attached to each other by their extremes. When the guard cells are turgid, due to their vacuoles being full of water, the pore opens (Figure 1A). When the vacuoles are emptied and water exits the cells, the guard cells become flaccid and the pore closes (Figure 1B) [1]. Loss of turgor pressure (and the resulting closure of the stomatal pore) is a consequence of the efflux of ions out of the cell. Ion efflux may be caused by a variety of stimuli including different light conditions and atmospheric carbon dioxide (CO_2_) levels, or signalling hormones such as abscisic acid (ABA) and ethylene [2,3]. Open pores allow the plant to absorb CO_2_ from the air to perform photosynthesis and to release oxygen and water into the atmosphere. If the pores close, the exchange of gas and water vapour slows down and photosynthesis stops. 

Regulation of stomatal closure is at the core of the ability of plants to adapt to light cycles and to changing external conditions or challenges [4]. A good understanding of signalling mechanisms in guard cells is thus important to characterise plant responses to the environment—a key aspect of the development of crops with enhanced productivity and improved resistance to drought, heat or pathogens. In addition, guard cells provide a prototypical platform for the study of cellular signal transduction since experiments are relatively accessible: changes in aperture are easy to observe and can be induced through changes in the concentration of different signalling molecules. General insights gained from such processes in guard cells could be of interest in other systems where the measurement of physiological responses is less straightforward [2,4-6].

### ABA-induced stomatal closure

**Figure 2:**
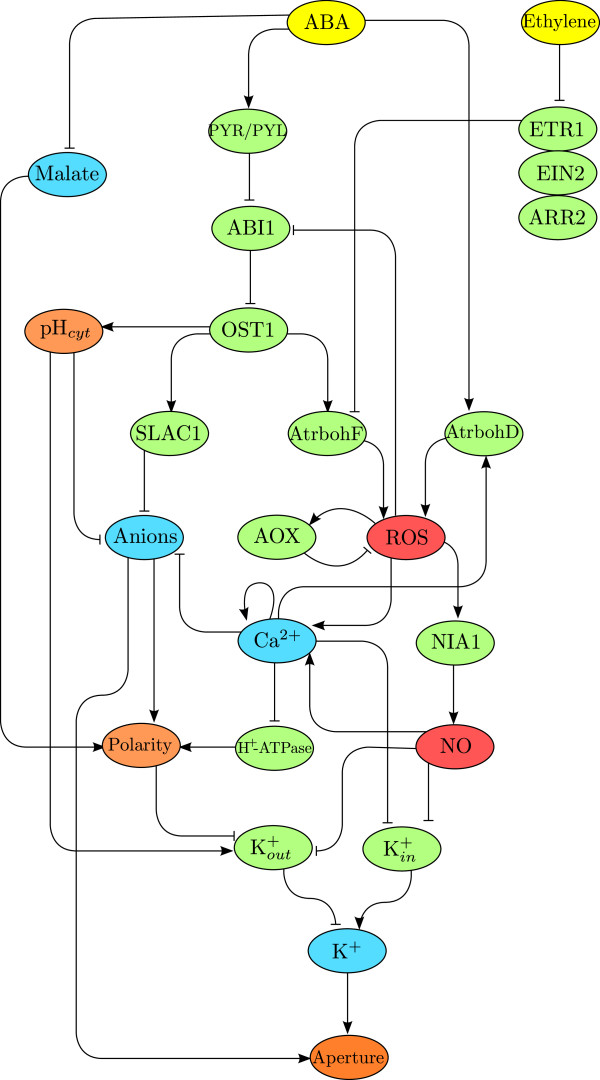
**Integrated ABA and ethylene signalling network in guard cells.** (Colour) Interactions between the components of the network are shown by the lines that join them. Positive interactions such as activation or production are represented with lines that end in an arrowhead →. Negative interactions such as inactivation, repression, or scavenging are represented by lines that end in a hammerhead ⊣. Yellow nodes are hormones, green nodes; proteins, blue nodes; ions, red nodes; reactive molecules, and orange nodes; physical properties of the cells. The interactions shown in this network (explained in the main text) are obtained from several previously published reports; however, this is the first time that the ABA and ethylene pathways appear together.

Abscisic acid is a hormone involved in a variety of key physiological processes in plants. In particular, ABA is a stress signal that induces stomatal closure in response to drought. We now review briefly some of the relevant biology of the ABA signalling network in guard cells, specifically in relation to redox processes (see Figure 2 for a schematic summary).

The characterisation of ABA receptors in guard cells is an active area of research [2,5,7-9]. Recently, ABA receptors of the pyrabactin resistance (PYR) and PYR-like (PYL) families have been identified in guard cells [10,11]. ABA-bound PYR/PYL/RCAR proteins can sequester the protein phosphatase 2C ABA-insensitive 1 (ABI1), preventing it from dephosphorylating the kinase open-stomata 1 (OST1), inducing the downstream response [12-14]. It is worth noting that there are other ABA receptors involved in stomatal closure, including the Mg-Chelatase H-subunit (CHLH) and two G-protein coupled receptor type G-proteins (GTG1 and GTG2) [15,16]. However, the precise interaction of these receptors with the rest of the ABA network in Figure 2 has not yet been clearly established and the role of CHLH as an ABA receptor has even been disputed [17].

Due to the ABA-induced sequestration of ABI1, OST1 is free to phosphorylate the NADPH-oxidase *Arabidopsis thaliana* respiratory burst oxidase homolog F (AtrbohF), which produces superoxide followed by quick dismutation to give hydrogen peroxide (H_2_O_2_) [18-20]. Superoxide and hydrogen peroxide are reactive oxygen species (ROS) [21] and for the remainder of this work we use the term ROS to signify hydrogen peroxide and associated species. Furthermore, ROS have been reported to inhibit the phosphatase activity of ABI1 *in vitro*, which could lead to the potential creation of a positive feedback loop [22]. These transduction steps indicate that an increase in the application of ABA leads to an increase in the production of ROS. We remark that this pathway is not the only mechanism through which ABA induces the production of ROS. For instance, an uncharacterised activation mechanism involving another NADPH-oxidase homolog, AtrbohD, is also active in ABA-induced stomatal closure, although of less importance in this process, as shown by mutant studies [19]. Rises in ROS production are controlled by anti-oxidants, such as ROS scavengers glutathione [23] and ascorbate [24], in order to avoid damage from oxidative stress [25].

ABA is also involved in the induction of other cellular responses. In particular, ABA activates vacuolar proton pumps (V-ATPases) which elevate the cytosolic pH (*pH*_*cyt*_) from about 7.0 to 7.5 and decrease the vacuolar pH (*pH*_*vac*_) from 5.5 to 5.0 [9,26-31], in a process that involves OST1 [32]. Conflicting reports about the involvement of ROS in the rise of *pH*_*cyt*_ suggest a complex interaction between them: in Ref. [32] alkalinisation of the cytosol is reported to precede ROS production, whereas Ref. [31] reports that *pH*_*cyt*_rises in response to ROS elevation. ABA signalling also leads to increased production of nitric oxide (NO) via the nitrate reductase 1 (NR1 or NIA1) in a process that requires ROS [33-35].

Increased *pH*_*cyt*_ and NO concentration cause a reduction in the concentration of K^+^ ions in the cytosol through an increased efflux and a reduced influx: the rise in *pH*_*cyt*_ increases the number of available outwards-rectifying K^+^ channels (*I*_*K*,*out*_) which accelerate K^+^ ion extrusion [28,36], while increased levels of ROS and NO promote the release of Ca^2+^ from its intracellular stores [37], leading to down-regulation of inwards-rectifying K^+^ channels (*I*_*K*,*in*_) [38-40]. It is also believed that ABA signals ‘prime’ Ca^2+^ receptors to make them more sensitive to cytosolic Ca^2+^ concentration and to ensure that the closure signal gets through [37]. However, higher concentrations of NO (≥100nM) can block *I*_*K*,*out*_directly by nitrosylation [38,41,42]. Elevated ROS concentrations can also block *I*_*K*,*in*_[43] and, surprisingly, *I*_*K*,*out*_although here the blocking of outwards K^+^ channels by ROS is probably not a prominent feature in ABA-mediated stomatal closure [44].

Other ions also play significant roles during ABA signalling. Anions such as chloride (Cl^-^) and malate are released from the vacuole and out of the cell during ABA-induced stomatal closure [45,46]. The kinase OST1 phosphorylates the protein slow anion channel-associated 1 (SLAC1), causing Cl^-^ efflux which depolarises the membrane [47]. Guard cells dispose of malate during stomatal closure by release and consumption through the tricarboxylic acid cycle [48]. A rise in cytosolic Ca^2+^ in response to elevated NO and ROS deactivates proton pump H^+^-ATPases which contributes to further depolarisation of the membrane [49] and thus allowing the outwards K^+^ channels (*I*_*K*,*out*_) to further increase its activity. The ion efflux and the resulting lower concentration of ions, anions and other solutes in the cytosol create an osmotic gradient that drives the transport of water from the vacuoles to the outside of the cell, leading to loss of turgor and to the closure of the stomatal pore [2,39]. The above information is summarised schematically in Figure 2.

### Ethylene-induced stomatal closure

The gaseous hormone ethylene is involved in diverse plant processes such as the control of root elongation, swelling of the hypocotyl and curvature of the apical hook (known as the triple response), as well as fruit ripening, leaf senescence, pathogen defence, and seed dormancy [1,50-52].

Ethylene is also an effector of stomatal closure [53]. Of the five known ethylene receptors, only ethylene receptor 1 (ETR1), which is located in the endoplasmic reticulum, has been shown to be involved in ethylene-induced stomatal closure [51,54-57]. During ethylene-induced stomatal closure, ROS are produced by AtrbohF (but not by its homolog AtrbohD) [58]. The mechanism that links ethylene perception by ETR1 and ROS production is still uncharacterised (e.g., the likely participation of the enzymes ABI1 and OST1 in this process remains to be confirmed). However, as ETR1 is a known negative regulator of ethylene signalling, it can be expected to inhibit the activation of AtrbohF. When ethylene binds to ETR1 the inhibition is removed, allowing AtrbohF to become activated. Stomata with ETR1 gain-of-function mutants *etr1-1* and *etr1-3* are both insensitive to ethylene. However, whereas *etr1-1* is insensitive to treatment with H_2_O_2_, the response of *etr1-3* to H_2_O_2_ closely resembles the response of wild-type stomata [54]. These experiments suggest a double role of ETR1 during ethylene-induced stomatal closure: one of perception of the ethylene signal (upstream of ROS), and one of signal-relay (downstream of ROS). Additionally, the role of ETR1 downstream of ROS has been shown to depend on on the enzymes ethylene insensitive 2 (EIN2) and *Arabidopsis thaliana* response regulator 2 (ARR2) [58].

Direct experimental confirmation of the downstream components of the ethylene signalling pathway is not yet available. Given that ROS are produced in response to ethylene by AtrbohF (as in ABA-induced stomatal closure), it is expected that the other components downstream of ROS in the ABA pathway (e.g., NO production, Ca^2+^ release and ion efflux) also lie downstream in the ethylene pathway, thus explaining the ion efflux from the cell in order to create the osmotic gradient that drives the loss of turgor leading to stomatal closure. For example, it has been shown that ethylene signals require Ca^2+^ to produce a response in other cell types [59]. We summarise in Figure 2 these components of the ethylene signalling network in guard cells in conjunction with the ABA pathway.

### ABA and ethylene cross-talk in guard cells

Hormone interactions in plant cells are common [50,60-62]. We have discussed above the key aspects of signal transduction for ABA and ethylene in guard cells and shown that both of these hormones cause stomatal closure independently. However, contrary to expectation, when applied simultaneously they fail to achieve full closure [56]. This counterintuitive observation implies the existence of cross-talk mechanisms which remain largely unexplored. In fact, components of the ethylene pathway such as EIN2 have been known to antagonise ABA signals in other cell types [63].

The signal transduction network in Figure 2, though partial and non-exhaustive, highlights the strong interconnection of both signalling pathways. Complex signalling systems can lead to unexpected and non-trivial behaviour, such as bistability, oscillations, and chaos [64-67]. In this work, we investigate the possible causes of the ABA-ethylene cross-talk in guard cells experimentally and theoretically. We measure ROS production and stomatal aperture in cells treated with single and combined stimuli and develop an ordinary differential equation (ODE) model of stomatal closure based on biochemical pathway information. Our experimental and mathematical results indicate that antioxidant mechanisms play a key role in the lack of stomatal closure when guard cells are subjected to the combined stimulus of both hormones.

## Results

### Time course measurements of ROS and stomatal closure

Most data currently available in the literature measure stomatal closure in response to a stimulus at a particular time after treatment (typically within one to two hours) [34,68-70]. In contrast, it is often assumed that ROS production in guard cells occurs in a sharp burst [9,30], so that measurements of ROS are conventionally recorded only up to a short time after stimulation. A detailed temporal study of ABA or ethylene-induced signalling responses in guard cells is still lacking.

In order to improve our understanding of the signalling processes leading to the closing of stomata, we have carried out time course measurements of stomatal aperture and ROS concentration in *Arabidopsis* guard cells until 60 min after treatment. In particular, we track the temporal responses of guard cells to ABA and/or ethylene under single and combined doses of ABA and the ethylene precursor ACC (10 *μ*M) (see the Methods section in the Additional file 1 for a full description of the experimental methodology).

**Figure 3:**
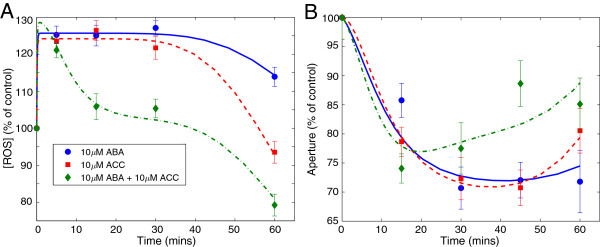
**Experimental data and modelling results.** (Colour) **A:** Experimental measurements of ROS in guard cells (markers with error bars) and solutions of equation (1) (lines) as percentage of control. **B:** Stomatal aperture measurements (markers with error bars) and solutions of equation (6) (lines) as percentage of control. On each image we show the response to 10 *μ*M ABA (blue circles), 10 *μ*M ACC (red squares), and 10 *μ*M ABA + 10 *μ*M ACC (green diamonds). Error bars show the standard error of the mean (*n*=30×3). The solutions to the equations were obtained using the parameter values from Table S1 in the Additional file 1.

The level of ROS production (relative to controls) in response to the treatments is shown in Figure 3A. ROS production is almost indistinguishable between all treatments up to five minutes after the stimuli, with an initial 25% increase in ROS concentration above control levels after 5 minutes. These increased levels were maintained until 30 minutes after treatment for cells stimulated with ABA or ethylene (ACC) alone. The ROS concentration then decreased in both cases. At 60 minutes, ROS levels in cells treated with ABA remained approximately 15% higher than control whereas ROS levels in cells treated with ethylene returned approximately to control levels. In the case of combined treatment with ABA and ethylene, the initial burst response was similar, with ROS levels increasing 25% after 5 minutes. However, this initial rise was followed by a rapid decrease to levels only 5% above control from 15–30 minutes and another further decrease to 80% of control at 60 minutes (Figure 3A).

The aperture responses of the guard cells treated with single and combined ABA and ethylene stimuli are shown in Figure 3B. All treatments produced a decrease in stomatal aperture within 15 minutes of treatment to varying degrees: the cells with the combined ABA-ethylene dose showed an aperture reduction of 25% compared to control, while cells treated with single stimulus of ABA and ethylene showed reductions of about 15% and 20%, respectively. After 30 minutes, the stomata with the combined treatment re-opened and continued to do so after 45–60 minutes. Cells treated with ABA maintained their apertures 30% below control from 30–60 minutes whilst apertures of cells treated with ethylene decreased to the same level (30% below control) from 30–45 minutes but had begun to re-open after 60 minutes, yet still remained below control.

The time profiles of ROS production and stomatal aperture indicate that a sustained elevation of ROS levels is needed to maintain stomata in a closed state, as seen by the decrease in aperture that coincides with the raised level of ROS up to 30 minutes with individual ABA or ethylene stimulation. Similarly, the decrease in ROS at 60 minutes under ethylene stimulation coincides with re-opening of stomata. In case of the combined stimulus, the large decrease in ROS coincides with re-opening of the stomata.

Our results confirm the importance of ROS in ABA and ethylene-induced stomatal closure, as described above and reported in Refs. [19,30,40,58]. Our experiments can also provide information about the mechanisms involved in guard cell signalling. In particular, Figure 3A suggests that there is an anti-oxidant mechanism, which is only active under the compound stimulus, that removes hydrogen peroxide from the cells. Although the particular mechanism at work has not been identified, cross-talk between the ABA and ethylene signalling pathways has been shown to occur [63]. Therefore, we hypothesise that these interactions of the ABA and ethylene pathways seem to induce a specific, rapid antioxidant activity which is not present under a single stimulus. This response is in addition to a general antioxidant response to sustained high levels of ROS present, which explains the observed decay of ROS after 30 minutes under all treatments (Figure 3A). One possible explanation is that the individual stimuli induce both stimulation of ROS production and a *delayed* antioxidant response to allow ROS-mediated signalling to occur, removing ROS only after the signal has been transmitted. This delay would be lacking under the combined stimulus situation, when another antioxidant mechanism is initiated early on.

The observations reported here suggest that complex interactions between the ABA and ethylene signalling pathways at the antioxidant level may be responsible for the reversal of stomatal closure following a combined stimulus. In the sections below, we explore this hypothesis further with the development of a mathematical model of signal transduction in relation to stomatal closure that incorporates the description of the antioxidant features we have just described.

### A model of signal transduction for stomatal closure

As previously discussed, unexpected outcomes may arise when combined stimuli are presented to a complex signalling network [56]. The ABA signalling network in guard cells has been studied computationally from a dynamic boolean network perspective [5,71] and the ethylene pathway in root cells has been modelled using ordinary differential equations (ODEs) [72,73]. However, to our knowledge, there are no models of stomatal closure that incorporate both ethylene and ABA.

In order to investigate the observed reversal of closure under a combined ABA and ethylene stimulus, we have developed a model of ODEs for the signal transduction of these inputs in connection with stomatal closure. Our choice of an ODE model is motivated by the small number of variables measured in our time course experiments; by the importance of the dynamical effects, as seen in Figure 3; and by the flexibility such a model affords us to include the biological interactions discussed above (Figure 2) in a simplified form. We have aimed for a simplified model with a small number of equations and parameters (relative to the actual number of known components of the guard cell signalling pathway), while still preserving dynamics and timescales. Such reduced models are useful to test alternative biological hypotheses and can be amenable to detailed analysis using bifurcation theory and comprehensive sensitivity analysis [74]. Given the lack of knowledge about the precise sequence of reactions and interactions among the members of the guard cell transduction pathways, we decided against larger models with many biochemical reactions and biophysical features. Furthermore, published observations on this system lack consistency across species, dose, time-points and experimental conditions, which would make the parametrisation of a large model impractical.

**Figure 4:**
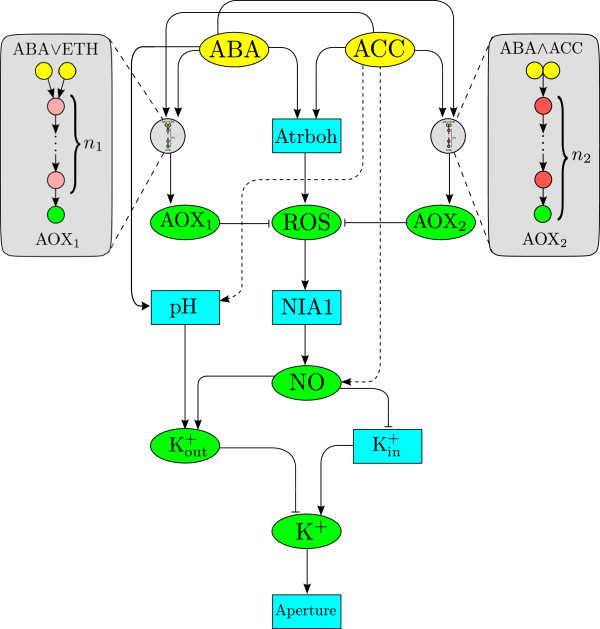
**Model of stomatal closure under ABA and ethylene stimuli.** (Colour) All the nodes in this picture are either explicitly or implicitly included in equations (1)-(6). The input nodes are shown as yellow ellipses, variable nodes are shown in green ellipses, and nodes that are blue rectangles are implicitly represented in the equations. The two circular grey nodes represent parts of the signal transduction network whose components are not yet known, and we include them as linear activation cascades (see text). The cascade AOX_1_ (on left) is activated by performing a logical *or* operation on the ABA and ethylene signals, the presence of either suffices to elicit a response. The cascade upstream of AOX_2_ (on right) becomes activated by performing a logical *and* operation on the ABA and ethylene signals; the presence of both of them is required to elicit a response. Dashed lines represent proposed pathways that require experimental verification.

We have constructed our model starting from descriptions of known interactions (Figure 2) which have then been simplified by the use of biologically motivated assumptions, described in detail in the Additional file 1. The model we present here was selected among several others representing different hypotheses (see the section on model selection in the Additional file 1) and describes the dynamics of six variables in terms of the external input functions *ABA* and *ACC*, which denote the doses of ABA and ethylene respectively. In addition to the experimentally measured *ROS*, we also describe the variable *NO*, which plays an essential role in stomatal closure by promoting the release of Ca^2+^ from stores and by mediating the blocking of inward rectifying K^+^ channels (I_*K*,*in*_) [38]. Following our experimental indications, we consider two different antioxidant mechanisms described by variables *AOX*_1_ and *AOX*_2_, which lie at the end of linear activation cascades driven by *ABA* and *ACC*. The variable $\left(\left[K_{\mathrm{out}}^{+}\right]\right)$ represents the active outwards-rectifying K^+^ channels *I*_*K*,*out*_while the variable *K*^ + ^ represents the concentration of potassium ions in the cytosol. We provide a separate equation for $\left(\left[K_{\mathrm{out}}^{+}\right]\right)$ in order to accurately incorporate the known interactions with NO and ABA. An explicit equation for $\left(\left[K_{\mathrm{in}}^{+}\right]\right)$ was not introduced because its numbers are believed to remain relatively constant during stomatal closure. The equations of the model are (Figure 4):

(1) $$\begin{array}{ll} \frac{d[\text{ROS}]}{dt}= & \alpha_{10}+\frac{\alpha_{11}k_{12}[\text{ABA}]+\alpha_{12}k_{11}[\text{ACC}]}{k_{11}k_{12}+k_{12}[\text{ABA}]+k_{11}[\text{ACC}]}\\ & -\left(\beta_{11}[\text{AO}X_{1}]+\beta_{12}[\text{AO}X_{2}]\right)[\text{ROS}], \end{array}$$

(2) $$\begin{array}{ll} \frac{d[{\text{AOX}}_{1}]}{dt}= & \alpha_{20}+\frac{\alpha_{21}k_{22}[\text{ABA}]+\alpha_{22}k_{21}[\text{ACC}]}{k_{21}k_{22}+k_{22}[\text{ABA}]+k_{21}[\text{ACC}]}\\ & \times P\left(n_{1},\alpha_{23}t\right)-\beta_{20}[\text{AO}X_{1}], \end{array}$$

(3) $$[\text{AO}X_{2}](t)=\frac{\left[\text{ABA}][\text{ACC}\right]}{\left(k_{11}+[\text{ABA}])(k_{12}+[\text{ACC}]\right)}P(n_{2},\beta_{13}t).$$

(4) $$\frac{d[\mathrm{NO}]}{dt}=\frac{\alpha_{31}[\text{ROS}]}{k_{31}+[\text{ROS}]}+\frac{\alpha_{32}[\text{ACC}]}{k_{12}+[\text{ACC}]}-\beta_{30}[\text{NO}],$$

(5) $$\frac{d[K_{\mathrm{out}}^{+}]}{dt}=\alpha_{40}+\frac{\alpha_{41}[\text{ABA}]}{k_{11}+[\text{ABA}]}+\alpha_{42}[\text{NO}]-\beta_{40}[K_{\mathrm{out}}^{+}],$$

(6) $$\frac{d[K^{+}]}{dt}=\frac{\alpha_{51}}{k_{51}+[\text{NO}]}-\beta_{50}[K_{\mathrm{out}}^{+}][K^{+}].$$

All the variables in equations (1)-(6) are expressed as percent of control, e.g., [*ROS*]=100 means that the amount of ROS in treated cells is equal to the amount of ROS in the control cells. It should also be noted that the stimulus is applied at time *t*=0, i.e., [*ABA*]=[*ACC*]=0 for *t*<0.

A brief description of the terms in the model is as follows: 

● Equation (1) describes the change of ROS concentration in the cell. The first constant term represents constant (endogenous) ROS production by processes unrelated to ABA and ethylene signalling. The second term describes ROS production in response to ABA and ethylene signals. Although two enzymes ( AtrbohF and AtrbohD) have been identified as sources of ABA-induced ROS in guard cells, only AtrbohF regulates ethylene-induced stomatal closure [19,58], and our data show similar initial increases in ROS upon an ABA or ethylene stimulus. Therefore, the contributions of the ABA-specific *AtrbohD* is at best marginal [19] and we have included only one ABA and ethylene-responsive ROS source, corresponding to AtrbohF (see Additional file 1). This second term has a compound Michaelis-Menten form that describes enzymatic activity in response to any of the two different signals (*ABA* or *ACC*). The final term represents the removal of ROS through reaction with the antioxidants *AOX*_1_ and *AOX*_2_ through a simple bimolecular reaction term.

● Equation (2) represents the change in antioxidant *AOX*_1_. The first term describes constant basal production. The second term describes the production of AOX_1_ by a cascade initiated by ABA *or* ethylene signals (Figure 4). The functional form of this term follows by assuming a linear cascade with an input given by a compound Michaelis-Menten term which performs a logic ‘or’ operation on the ABA and ethylene signals and can become saturated. This term also contains the normalised incomplete Gamma function *P*(*n*_1_*α*_23_*t*), which appears as the analytical solution of a linear activation cascade (see derivations in the Additional file 1 and Ref. [75]). Here, *n*_1_is a measure of the length of the cascade and the term introduces an effective delay in the production of antioxidants, enough to let the ROS signal be transmitted downstream. The last term is a standard linear decay or removal with characteristic constant *β*_20_.

● Equation (3) is the analytical solution of a linear cascade of length *n*_2_with input given by the product of two Michaelis-Menten terms. Therefore, this signal transduction pathway is only active when both *ABA*>0 and *ACC*>0, thus enforcing a logical ‘and’ operation on the signals. The hypothesis behind *AOX*_2_ follows from our experimental observation that when the cells are challenged with the two hormones they create an antioxidant response which is not active when there is only one stimulus present simultaneously (Figure 3). The Michaelis-Menten terms (with the same saturation constants as equation (1)) saturate the antioxidant response to the doses of ABA and ethylene. Logical *and* and *or* gates in biochemical systems can be the result of particular post-translational modification of enzymes (e.g., multiple phosphorylation, trimerisation, etc. See the discussion about the possibility of an allostery-driven mechanism in the Additional file 1) [76].

● Equation (4) describes the change in *NO*. The first term shows the production of NO by the enzyme NIA1, whose function depends on the presence of ABA-induced ROS [34]. The second term describes further NO production from ethylene, which could be either from NIA1 or another, yet unidentified source (see Additional file 1). The term has a Michaelis-Menten form to account for the fact that at least the perception of ethylene is mediated by an enzyme and can therefore saturate—the Michaelis constant *k*_12_is the same as in equation (1). The third term models NO removal, for which several mechanisms exist in plant cells [77].

● Equation (5) shows the change in $\left(\left[K_{\mathrm{out}}^{+}\right]\right)$, the active outwards K^+^ channels. The first (*α*_40_) and last ($\left(\beta_{40}[K_{\mathrm{out}}^{+}]\right)$) terms represent the constant flux of channels between the active and inactive states, respectively. The second term represents the extra number of channels made available by the increase in cytosolic pH (*pH*_*cyt*_) following an ABA stimulus. This term is a Michaelis-Menten form with the same constant *k*_11_as in equation (1). The third term (*α*_42_[*NO*]) is the increase in *I*_*K*,*out*_channel activity as a result of membrane depolarisation possibly via NO-induced Ca^2+^ release (i.e. via the path *NO*→*Ca*^2 + ^⊣*H*^ + ^-*ATPase*→*Polarity*⊣*I*_*K*,*out*_in Figure 2).

● Equation (6) shows the change in *K*^ + ^. The first term represents the increase of ions that enter through the inwards-rectifying channels (*I*_*K*,*in*_), which are inactivated by NO [38]. The second term is the ion efflux through the outwards channels that is proportional to the active channels $\left(\left[K_{\mathrm{out}}^{+}\right]\right)$ and the ion concentration itself.

● The relationship of this model to stomatal aperture is via the last variable *K*^ + ^. Cell volume (and hence stomatal aperture) is determined by the ion and solute concentration in the cell relative to the external concentration [57,78]. Therefore, ions and solutes (K^+^ in particular) can be taken as a simple proxy for aperture:

$$[\mathrm{AP}]=[K^{+}],$$

which is given as percentage of control.

● We have also considered in detail an alternative version of the model that considers the hypothesis that ethylene affects cytosolic pH instead of producing NO. This requires that *α*_32_=0 and equation (5) to be:

(7) $$\begin{array}{ll} \frac{d[K_{\mathrm{out}}^{+}]}{dt} & =\alpha_{40}+\frac{\alpha_{41}[\text{ABA}]}{k_{11}+[\text{ABA}]}+\alpha_{42}[\text{NO}]+\frac{\alpha_{43}[\text{ACC}]}{k_{12}+[\text{ACC}]}\\ & \;-\beta_{40}[K_{\mathrm{out}}^{+}]. \end{array}$$

This hypothesis has the same number of parameters as the model above and fits the data equally well (more about this model is found in the following sections and in the Additional file 1).

One of the distinctive aspects of the model is our approach towards the simplification of uncharacterised activation cascades. As discussed earlier, activation cascades can control the strength and timing of cellular responses [79]. In particular, it is key that the modelling of the cascades in this model incorporates a representation of the implicit delay present in the antioxidant response, which occurs in parallel to the production of ROS (Figure 4). In order to accomplish this parsimoniously within the setting of ODEs, equations (1) and (2) incorporate cascading terms which introduce a delay through the effect of sequential processes. The introduction of such terms is commonplace as an alternative to more complex delay equations [80,81]. In our case, we have used a simple model of a linear activation cascade with identical deactivation rates [79], which has been shown to provide optimal signal amplification [82]. Each cascading module has an explicit analytical solution in terms of the normalised incomplete gamma function and introduces only three parameters to the model [75].

Finally, it is important to remark that our model is constructed to represent only the signal transduction processes, i.e., the transient dynamics following different external inputs. We emphasise that we have not considered further downstream mechanisms that would dominate the dynamics at longer timescales. Stomatal closure in response to either ABA or ethylene is a relatively fast process that takes place in time scales shorter than typical genetic regulation. Maintaining stomatal closure and inhibiting stomatal opening are separate processes [15], and require other regulatory interactions and expression of certain genes [83] which we do not consider here. Therefore, we are only concerned in this work with short-term, transient behaviour of stomata and we do not study the stationary dynamics of the model.

### Numerical results from the model

#### Parameterisation of the model: fitting to experimental observations

The model of signal transduction (1)–(6) has six variables and consists of five coupled nonlinear ODEs (one of the variables can be solved explicitly as a function of time) with 28 parameters. We fit the model to experimental data using the *Squeeze-and-Breathe* algorithm [84], a recent optimisation method that can efficiently fit temporal data using an accelerated Monte Carlo search and fit process (see Methods section). The parameters of the model found using the Squeeze-and-Breathe algorithm are shown in Table S1 of the Additional file 1. The fits to the data in Figure 3 are shown to match the observations, specifically the response to the combined stimuli.

#### Dynamical response of the model to stimuli and model verification

**Figure 5:**
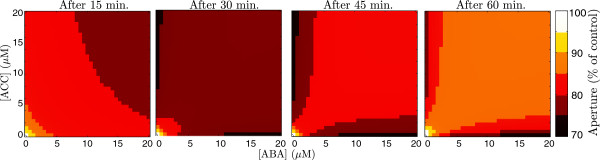
**Map of model responses to treatment combinations.** Predicted aperture response map of combinations of ABA and ACC doses 15, 30, 45, and 60 minutes after treatment. The coordinates of each point in represent the dose combination and the colour denotes the response. Dark regions indicate less aperture (i.e., more closure in response to treatment) and lighter regions show more aperture. Response apertures shown as percent of control.

**Figure 6:**
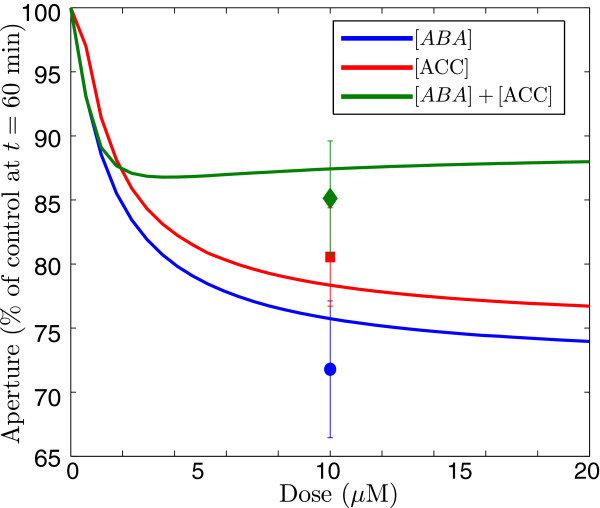
**Predicted aperture response to combinations of ABA and ethylene doses at 60 minutes.** (Colour) Dose response of the aperture individual doses of ABA (blue line) and ACC (red line), which are the responses along the *x* and *y* axes of the far-right image of Figure 5. The green line is the response to the combined stimulus of equal doses of ABA and ethylene, which corresponds to the values along the diagonal in the heat map. The data points are the experimental measurements at *t*=60 minutes of the aperture shown in Figure 3.

The dynamical behaviour of the model of signal transduction is explored in Figures 5 and 6. The heat maps in Figure 5 show snapshots of the time course of stomatal aperture following treatments of different dose combinations of ABA and ethylene, represented on the (*ABA*, *ACC*) plane. The simulations reproduce the observation that doses of combined treatment result in diminished closure (that could lead to enhanced aperture if enough ROS is depleted) over the time course, as compared to the increased closure induced by doses of single treatments. The variables respond with different intensity to the treatments. In particular, the model predicts that ethylene has a stronger *AOX*_1_ response than ABA, to reflect the differences observed in Figure 3A. The antioxidant *AOX*_2_ is only active during treatment with both hormones, as previously discussed. Figure 6 shows that the responses to the two hormones (after 60 minutes) are asymmetrical, with ABA inducing more closure than ethylene. This asymmetry of the response is consistent with reports in the literature that stomata respond more strongly to ABA than to ethylene [56], though it appears to diminish with increased doses. 

**Figure 7:**
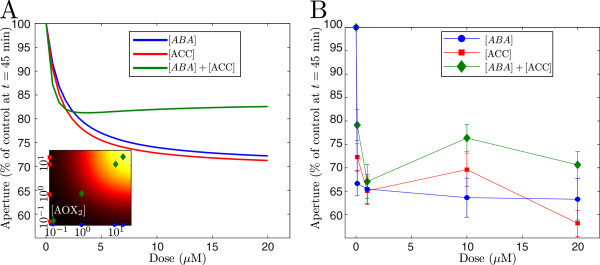
**Comparison of model predictions to data at 45 minutes.** (Colour) **A**: Dose responses predicted by the model to different doses of ABA (blue line), ACC (red line), and a combination of both (green line), 45 minutes after the stimulus. *Inset*: Response of AOX_2_ at *t*=45 to the dose combinations between 0.1*μ*M and 30*μ*M ABA and ACC. The markers are there to indicate the response of AOX_2_ to the treatments used on the right image. **B**: Dose responses observed in experimental measurements of stomatal closure 45 minutes after single and combined doses of 0.1*μ*M, 1.0*μ*M, 10*μ*M, and 20*μ*M ABA and ACC (*n*=30×3, these data were not used to re-parametrise the model).

A prediction of the model is that high doses of the combined stimulus ‘backfire’ past 45 minutes resulting in the stomata re-opening rather than maintaining closure. The re-opening of the stomata in our model is a direct consequence of the excess antioxidant activity (i.e., [*AOX*_2_]) that results from the interaction of the ABA and ethylene pathways. Figure 7 compares the dose responses predicted by the model after 45 minutes (Figure 7A) to experimentally observed responses to single and combined 0.1*μ*M, 1.0*μ*M, 10*μ*M, and 20*μ*M ABA and ACC stimuli (Figure 7B). We note that these measurements were not used to re-parametrise the data. The data, though noisy (an inherent feature of stomatal assays), are qualitatively reflected by the behaviour predicted by the model. The combined treatments of 0.1*μ*M and 1.0*μ*M ABA and ACC show a similar response to the single treatments. These small doses are sufficient to trigger some closure but not strong enough to activate a response by AOX_2_ (inset of Figure 7A). The stronger combined treatments (10*μ*M and 20*μ*M) are enough to activate AOX_2_ which at *t*=45 already has begun to reverse closure.

Another feature emerging from our model is the identification of two relevant time scales associated with the two anti-oxidant responses. The fitted parameters in the cascade terms in equations (2) and (3) can be related to time delays *τ*_1_ and *τ*_2_ that lead to the activation of AOX_1_ and AOX_2_, respectively:

$$\tau_{1}=\frac{n_{1}}{\alpha_{23}}-1\approx 111\;\min,\;\tau_{2}=\frac{n_{2}}{\beta_{13}}-1\approx 12\;\mathrm{min.}$$

These time scales (an order of magnitude apart) point at qualitatively different biological mechanisms behind the observed antioxidant effects: the time scale of the response of the ‘or’ antioxidant (AOX_1_) suggests a transcriptional response whereas the time scale of the response of the ‘and’ antioxidant (AOX_2_) is much faster and its action is unlikely to depend on gene expression, but rather on post-translational modifications or direct enzyme activation. Furthermore, our sensitivity analysis shows that *α*_23_ and *β*_13_ are the most sensitive parameters in the model (see Additional file 1) which indicates that the timescales of the antioxidants are crucial in the regulation of stomatal movements. These predictions suggest that ABA or ethylene alone stimulate gene expression which may be required for ROS-induced maintenance of stomatal closure. However in the presence of ABA and ethylene other non-transcriptional, rapid mechanisms activate antioxidant machineries that allow stomata to remain open.

## Discussion and conclusions

In this work, we have investigated experimentally and theoretically why full stomatal closure fails to occur when guard cells are presented with a combined ABA-ethylene stimulus, an observation first reported in Refs. [56,58]. As shown in Figure 2, the pathways of both hormones overlap strongly, with ROS playing a significant role. Our time course measurements of ROS and aperture over 60 minutes in guard cells under single stimuli (ABA, ethylene) and combined stimulus (ABA plus ethylene) show that when both hormones are present, ROS are removed swiftly after an initial burst of production and the closure process reverses. This is the first report of a rapid change and shift in pattern of ROS production in guard cells depending on the type and number of input stimuli.

In order to gain insight into the process of ROS removal, we have developed an ODE model of signal transduction leading to stomatal closure. Based on the experimental data, our model posits the existence of two separate antioxidant mechanisms active in guard cells. Firstly, a generic antioxidant mechanism operational in response to a single ROS-generating stimulus (ABA *or* ethylene) that allows ROS to signal downstream and then removes ROS to control oxidative stress on a time scale of around 2 hours. Secondly, an antioxidant response active only when both hormones (ABA *and* ethylene) are present simultaneously which does not allow the ROS signal to persist long enough to maintain closure, thus disrupting the closure process. This second response occurs on the time scale of around 10 minutes. The difference in the timescales of each of the antioxidant responses suggests the possibility that the generic ‘or’ mechanism (AOX_1_) requires a transcriptional response, whereas the combined ‘and’ response (AOX_2_) does not. As mentioned above, guard cells have a variety of antioxidant mechanisms, some of which are enzymatic, such as ascorbate peroxidase, superoxide dismutase (SOD) and catalase (CAT), and others are non-enzymatic, including glutathione (GSH), ascorbate (ASC), tocopherol, flavonoids, carotenoids and NO [23,24,85-87]. Determining the identity of AOX_1_ and AOX_2_ and their precise mechanism of action remains an important task for understanding stomatal response to multiple stimuli.

Our modelling also suggests that ethylene could have more than one pathway to produce NO and/or be able to elevate cytosolic pH levels. In particular, an alternative version of our model with a modified term in equation (5) to represent ethylene-induced cytosolic alkalinisation (see Additional file 1) is equally able to represent the dynamics of guard cells reported here. Simulations on models without a ROS-independent pathway from ethylene to NO (i.e., simulations on models where NO is exclusively produced via ROS) *and* where ethylene does not have an effect on pH, do not reproduce the response that we report here. Similarly with the active *I*_*K*,*out*_ channels: ABA-driven alkalinisation alone is not enough to create the outwards flux of ions needed to achieve stomatal closure, when an NO term is added to equation (5) the necessary ion flux for stomatal closure is attained. The relationship between NO and *I*_*K*,*out*_ is unlikely to be direct, although NO can block *I*_*K*,*out*_ by nitrosylation [41]. Enhancement of *I*_*K*,*out*_activity by NO is more likely driven by membrane depolarisation in response to Ca^2+^ release or cytosolic alkalinisation (Figure 2, see Additional file 1). In ongoing parallel experimental studies in our lab, we are uncovering new signalling pathways downstream of ethylene that appear to be ROS-independent, and we are investigating components of the ethylene pathway beyond ROS.

Our experimental and modelling results point towards the action of antioxidants as the cause for the reversal of stomatal closure under a combined ABA/ethylene treatment. Although these antioxidants have not been identified yet, there are components of the pathway that are known to have antioxidant activity such as NO which has been shown to react with superoxide [77], to enhance dessication tolerance [88], and to nitrosylate NADPH-oxidase [89]. Although the interactions between ABA, ethylene, signalling molecules and antioxidants are highly complex, our work suggests that ROS production and removal is tightly linked to stomatal closure in guard cells. Our results also put forward the possibility that ethylene may have a ROS-independent way of producing NO and/or increase cytosolic pH, with effects on Ca^2+^ and membrane polarity that need to be elucidated.

We have also considered the possibility of a biochemical basis for the observed response to the compound stimulus. Following a cue from Ref. [90] where AtrbohF was found to have two phosphorylation sites, we have explored whether an allosteric effect may be responsible for the ROS-deficit under a combined stimulus. The idea is that AtrbohF could be independently activated at different sites by single treatments of ethylene and ABA (something that has not been established experimentally) leading to the production of ROS and closure, while simultaneous signals would result in a doubly-phosphorylated AtrbohF unable to produce ROS. Then one would potentially expect to observe similar behaviour as the one reported here. We have tested this idea and found that in fact it is not able to reproduce the temporal dynamics of our ROS observations in Figure 3A (see Additional file 1).

Our model predicts that a combined stimuli of ABA and ACC above a critical level results in the arrest of the closure process (Figures 5, 6 and 7), a consequence of the failure to maintain the increased ROS production necessary for successful closure due to increased antioxidant activity. Additional experiments have shown that stomatal closure does occur for combined treatments provided the doses are low enough (Figure 7B), though the response to stronger doses suggest a maintaining of opening status. It must be noted that a detailed description of ABA/ethylene receptor dynamics is not included in our model, as there are no clear data available for all receptors (this is specially true for ethylene receptors in guard cells). Future work will concentrate on establishing the identity of the antioxidants active during stomatal closure, and understanding signalling events downstream of ROS. More detailed experimental observations will be instrumental in the next iteration in the development of improved models with greater predictive power.

In addition, further research will be necessary to ascertain if the physiological concentrations of both ABA and ethylene present during environmental stimuli, such as bacterial challenge or high humidity that cause stomata to open [91,92] fall within the ranges tested experimentally in our work. In the natural environment, plants face threats from multiple stimuli. Yet single stimuli are most often studied under laboratory conditions. This is partly due to the complexity and variability in responses that ensue following exposure to multiple stresses. Using guard cells as a model system we have considered mechanisms for a non-trivial output under a combination of stimuli. This study is a first step towards quantitation of a fundamental physiological process in plants, which is essential for growth and development.

## Methods

A detailed description of our experiments (including stomatal and fluorescence assays), modelling methodology, parameter fitting, model selection, sensitivity analysis, and simulations is included in the Additional file 1 that accompanies this paper.

## Endnotes

This paper is dedicated to the memory of Jaroslav Stark.

## Competing interests

The authors declare that they have no competing interests.

## Authors’ contributions

Research design: RD, MB, and MBD. Analysis and modelling: MBD. Experiments: MCHG, AML, RD, MBD. Writing: MBD, RD, MB. All authors read and approved the final manuscript.

## Supplementary Material

Additional file 1 Detailed information about experimental methods, the construction of the stomatal closure model, parameter fitting, activation cascades, and brief exploration of an alternative mechanism of ABA and ethylene cross-talk upstream of ROS production, model selection, and sensitivity analysis. Click here for file
