# Supplementary material for: Compound stress response in stomatal closure: a mathematical model of ABA and ethylene interaction in guard cells
Source: BMC Syst Biol. 2012 Nov 25;6:146. doi: 10.1186/1752-0509-6-146 (PMC3564773; doi:10.1186/1752-0509-6-146)
Supplement: Additional file 1 — Detailed information about experimental methods, the construction of the stomatal closure model, parameter fitting, activation cascades, and brief exploration of an alternative mechanism of ABA and ethylene cross-talk upstream of ROS production, model selection, and sensitivity analysis. [file 1752-0509-6-146-S1.pdf]

Compound stress response in stomatal closure: a mathematical model of ABA and Ethylene interaction in guard cells: Supplemental Information

M. Beguerisse-Díaz [1,2]*, M. C. Hernández-Gómez[1], A. M. Lizzul[1],
M. Barahona [2]†, and R. Desikan [1]‡
[1]Department of Life Sciences, [2]Department of Mathematics,
Imperial College London. London SW7 2AZ, U.K.

November 20, 2012

Contents

1 Materials and Methods **2**
1.1 Plant material . . . 2
1.2 ROS fluorescence assays . . . 2
1.3 Stomatal assays . . . 2

2 Construction of the stomatal closure model **3**
2.1 Signal perception and ROS production . . . 3
2.2 NO production . . . 7
2.3 Ca^{2+} increase, cytosolic alkalinisation, and ion efflux . . . 8
2.4 Loss of turgor . . . 9
2.5 A model of signal transduction for stomatal closure . . . 10
2.6 Alternative hypothesis: Ethylene-induced cytosolic alkalinisation . . . 10
2.7 Allostery hypothesis for ABA-ethylene cross-talk in ROS production . . . 11

3 Modelling of activation through linear cascades **11**

4 Compound Michaelis-Menten forms **13**

5 Parameter fitting **14**

6 Model selection **16**

7 Sensitivity analysis **19**

*m.beguerisse@imperial.ac.uk
†m.barahona@imperial.ac.uk
‡r.desikan@imperial.ac.uk

1 Materials and Methods

1.1 Plant material

Arabidopsis thaliana seeds of Columbia ecotype (Col-0) were sown on Levington F2+S soil and grown under constant conditions in a growth chamber (Sanyo Gallenkamp, Loughborough, UK) with a 10 hour photoperiod a light intensity of $100 - 150\mu E/m^2/s$, $22°C$ and 70% of relative humidity. After 7 days, seedlings were transplanted individually into new pots and were maintained in the same conditions previously detailed. Leaves of 4-6 weeks old plants, which had not yet formed flower bolts, were harvested for the aim of these experiments.

1.2 ROS fluorescence assays

Stomatal H_2O_2 concentration was measured as described in Ref. [8]. Leaves from 4 weeks old plants (approximately 2 leaves per condition) were blended in deionised water and epidermal fragments were collected with a $40\mu m$ sterile cell strainer (Fisher Scientific). Epidermal fragments were incubated in Petri dishes containing MES/KCl buffer ($5mM$ KCl, 50 μM $CaCl_2$, $10mM$ MES buffered to pH 6.15 with KOH) inside the growth chamber for 3 hours. This allowed the stomata to open prior to the application of the treatments. Epidermal fragments were collected and equally distributed into Petri dishes loaded with ethanol (as control), $10\mu M$ ABA (2-cis, 4-trans abscisic acid 98%, synthetic, Aldrich), $10\mu M$ the ethylene precursor ACC (1-aminocyclopropane-carboxylic acid hydro-chloride, Sigma), and a combination of $10\mu M$ ABA and $10\mu M$ ACC. The treatments, which had durations of 5, 15, 30, and 60 minutes, were performed. Following treatment for the appropriate time, fragments were incubated with $50\mu M$ $H_2DCF - DA$ (2, 7 -dichlorodihydrofluorescein diacetate, Invitrogen) for 10 minutes for H_2O_2 detection. After a washing step in MES/KCl buffer for 20 minutes, epidermal fragments were placed onto a slide and observed under a microscope. All steps were carried out under dark conditions, as the dye is light-sensitive. H_2O_2 was visualised with a fluorescence microscope (Axioskop2 plus, Carl Zeiss Ltd., UK) with Zeiss filter set 3 (excitation light filter: 450-490nm, emission light filter: 515-565nm). Images were captured with Axiovision software v3.1 (Carl Zeiss Vision GmbH, UK). Images were processed and fluorescence intensities (as mean of the pixel intensities) were measured with ImageJ software [1].

1.3 Stomatal assays

Stomatal aperture bioassays were performed on 4 week old leaves, as described in Ref. [8]. Leaves from 4-6 week-old plants were then cut from the plants using scissors. Excised leaves were floated for 3 hours inside the growth chamber in Petri dishes with buffer ($5mM$ KCl, $50\mu M$ $CaCl_2$, $10mM$ MES, buffered to pH 6.15 with KOH). After the initial treatment in buffer the leaves were exposed to $1\mu M$, $10\mu M$ ABA, $1\mu M$, $10\mu M$ ACC, a combination of $10\mu M$ ABA with $10\mu M$ ACC, and ethanol as a control. The treatments were left in the growth chamber for 15, 30, 45 and 60 minutes. Two leaves were blended in water for 1-2 minutes and epidermal fragments collected on a $100\mu m$ nylon mesh (Spectra-Mesh, BDH-Merck, Nottingham, UK) and transferred to a microscope slide. Measurements of individual stomatal aperture were conducted (see Fig. 1 of the main text) using a Leica DME light microscope, connected to a Leica DFC290 camera imaging system (Leica, Milton Keynes, UK). Leica QWinV3 software (Leica QWIN software, Leica, Milton Keynes, UK) was used to measure the apertures. Data for each data point are the mean of 3×30 measurements.

2 Construction of the stomatal closure model

In this section we describe in detail the steps and assumptions taken to construct the ODE model of stomatal closure under single and combined ABA and ethylene stimuli.

2.1 Signal perception and ROS production

In the main text we describe the events in ABA and ethylene perception and production of ROS. These early events are represented in Fig. S1A by the subnetwork comprised of the nodes ABA, Ethylene, PYR/PYL, ABI1, OST1, AtrbohD/F, ROS, AOX, and ETR1, whose concentrations we denote by $[ABA]$, $[ACC]$, $[PYR]$, $[ABI1]$, $[OST1]$, $[AtrbohF]$, $[AtrbohD]$, $[ROS]$, $[AOX]$, and $[ETR1]$. Given the timescales of stomatal closure (up to 60 minutes), we do not consider gene expression so the total amount of each enzyme remains constant, thus we have the following conservation relations:

$$\begin{aligned}
[PYR_T] &= [PYR] + [PYR\text{-}ABA] + [PYR\text{-}ABI1],\\
[ABI1_T] &= [ABI1] + [ABI1\text{-}PYR],\\
[OST1_T] &= [OST1] + [OST1_P],\\
[AtrbohF_T] &= [AtrbohF] + [AtrbohF_P],\\
[AtrbohD_T] &= [AtrbohD] + [AtrbohD_P],\\
[ETR1_T] &= [ETR1] + [ETR1\text{-}ACC],
\end{aligned}$$

where a subscripted T indicates total concentration of an enzyme (a non-negative constant in $\mathbb{R}$), a subscripted P indicates phosphorylation (or, more generally, being active), and variable names joined with a dash are complexes. For example, the constant $[PYR_T]$ is the total amount of the ABA receptor PYR/PYL in any form, the variable $[PYR]$ is the concentration of "available" PYR/PYL molecules, $[PYR\text{-}ABA]$ is the concentration of ABA-bound PYR/PYL molecules, and $[PYR\text{-}ABI1]$ is the concentration of ABI1-bound PYR/PYL. We describe the events known to occur between ABA and ethylene perception and ROS production with the following ODE model:

$$\frac{\mathrm{d}[PYR\text{-}ABA]}{\mathrm{d}t} = k_1([PYR_T]\text{-}[PYR\text{-}ABA] + [PYR\text{-}ABI1])[ABA] - (k_{-1} + k_2([ABI1_T] - [ABI1\text{-}PYR]))[PYR\text{-}ABA], \tag{S1}$$

$$\frac{\mathrm{d}[PYR\text{-}ABI1]}{\mathrm{d}t} = k_2[PYR\text{-}ABA]([ABI1_T] - [PYR\text{-}ABI1]) - k_3[PYR\text{-}ABI1], \tag{S2}$$

$$\frac{\mathrm{d}[OST1_P]}{\mathrm{d}t} = k_4([OST1_T] - [OST1_P]) - (k_{-4} + k_5([ABI1_T] - [PYR\text{-}ABI1]]))[OST1_P], \tag{S3}$$

$$\frac{\mathrm{d}[AtrbohF_P]}{\mathrm{d}t} = k_6([AtrbohF_T] - [AtrbohF_P])[OST1_P] - (k_7 + k_8([ETR1_T] - [ETR1\text{-}ACC])[AtrbohF_P], \tag{S4}$$

$$\frac{\mathrm{d}[AtrbohD_P]}{\mathrm{d}t} = k_9([AtrbohD_T] - [AtrbohD_P])[ABA] - k_{10}[AtrbohD_P], \tag{S5}$$

$$\frac{\mathrm{d}[ETR1\text{-}ACC]}{\mathrm{d}t} = k_{11}([ETR1_T] - [ETR1\text{-}ACC])[ACC] - k_{12}[ETR1\text{-}ACC], \tag{S6}$$

$$\frac{\mathrm{d}[ROS]}{\mathrm{d}t} = k_{13} + k_{14}[AtrbohF_P] + k_{15}[AtrbohD_P] - (k_{16} + k_{17}[AOX])[ROS], \tag{S7}$$

$$\frac{\mathrm{d}[AOX]}{\mathrm{d}t} = k_{18} + k_{19}[ROS] - k_{20}[AOX]. \tag{S8}$$

Equations (S1)-(S3) describe the events of ABA perception and ABI1-phosphatase inhibition [12, 18], and OST1 phosphorylation [15]. Equations (S4) and (S5) describe the activation of AtrbohD/F where OST1 phosphorylates AtrbohF, unbound ETR1 inactivates AtrbohF, and AtrbohD is activated by ABA [8, 11]. Equation (S6) shows ethylene binding and subsequent inactivation of ETR1 [6, 7]. Equation (S7) shows the production of ROS by AtrbohD/F and by other

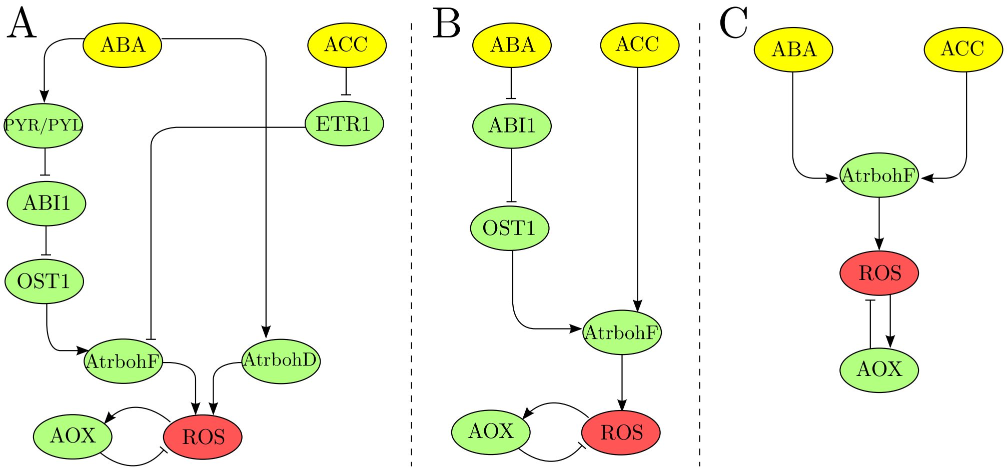

Figure S1: Simplification of signal perception and ROS production model. **A**: Network representation of the ROS production model (S1)-(S8). **B**: Simplification of the ROS production model given in equations (S9)-(S13). **C**: Further simplification of ROS production, given in equations (S15)-(S17).

cellular processes (summarised in the constant rate k_{13}), scavenging by antioxidants (summarised in the variable AOX), removal or decay of ROS ($k_{16}[ROS]$); equation (S8) shows that AOX has endogenous production (k_{18}), production in response to ROS ($k_{19}[ROS]$), and decay or inactivation ($k_{20}[AOX]$).

There are some things to note about the model above: the way in which the treatments are given (floated on a Petri dish, see Materials and Methods) allows us to assume that the concentration of ABA and ethylene does not change, hence $[ABA]$ and $[ACC]$ are constants. The amount of ATP for the phosphorylation reactions and of NADPH for the production of ROS are also considered abundant enough within guard cells and they are implicitly included in the parameters of the model. We assume that when the complex PYR-ABA-ABI1 dissociates, it does so completely (i.e., into PYR, ABA, and ABI1), to make the equations simpler; we also assume that unbound ETR1 negatively interacts with AtrbohF (or an upstream activator). For now, the variable $[AOX]$ clusters together the group of antioxidants that are active during stomatal closure. The model described in (S1)-(S8) has 8 equations and 20 kinetic parameters plus 6 parameters representing the total amount of the enzymes involved, which makes the task of determining parameter values from the ROS measurements a rather difficult one. We reduce the number of equations and parameters as much as possible by making a series of assumptions, but avoiding oversimplification of the system.

As noted in Refs. [8, 11], AtrbohD has a limited role in ABA-induced stomatal closure and none in ethylene-induced closure, therefore we assume that ROS is exclusively produced by AtrbohF. This assumption is supported by our ROS measurements which show similar initial increases in ROS upon an ABA or ethylene stimulus (main text, Fig. 3), for if there were two sources of ROS the pattern of initial increase would most likely differ. We use the quasi-steady-state assumption (QSSA) on the dynamics of the ABA and ethylene receptors, so equations (S1) and (S6) are assumed to reach equilibrium before the other variables have changed significantly. Under the QSSA, the expressions for the ligand-bound receptors become:

$$[PYR\text{-}ABA] = \frac{([PYR_T] - [PYR\text{-}ABI1])[ABA]}{\frac{k_{-1}}{k_1} + \frac{k_2}{k_1}([ABI1_T] - [PYR\text{-}ABI1]) + [ABA]},$$
$$[ETR1\text{-}ACC] = \frac{[ETR1_T][ACC]}{\frac{k_{12}}{k_{11}} + [ACC]}.$$

The expression for $[ETR1\text{-}ACC]$ is a standard Michaelis-Menten term with a maximum rate given by the total amount of ETR1. The expression for $[PYR\text{-}ABA]$ is a Michaelis-Menten-type term with a dependency on the variable $[PYR\text{-}ABI1]$ (which holds PYR/PYL molecules). The system in (S1)-(S8) becomes

$$\frac{\mathrm{d}[PYR\text{-}ABI1]}{\mathrm{d}t} = \frac{k_2([ABI1_T] - [PYR\text{-}ABI1])([PYR_T] - [PYR\text{-}ABI1])[ABA]}{\frac{k_{-1}}{k_1} + \frac{k_2}{k_1}([ABI1_T] - [PYR\text{-}ABI1]) + [ABA]} - k_3[PYR\text{-}ABI1], \tag{S9}$$

$$\frac{\mathrm{d}[OST1_P]}{\mathrm{d}t} = k_4(OST1 - [OST1_P]) - (k_{-4} + k_5([ABI1_T] - [PYR\text{-}ABI1]]))[OST1_P], \tag{S10}$$

$$\frac{\mathrm{d}[AtrbohF_P]}{\mathrm{d}t} = k_6([AtrbohF_T] - [AtrbohF_P])[OST1_P] - \left(k_7 + k_8[ETR1_T]\left(1 - \frac{[ACC]}{\frac{k_{12}}{k_{11}} + [ACC]}\right)\right)[AtrbohF_P], \tag{S11}$$

$$\frac{\mathrm{d}[ROS]}{\mathrm{d}t} = k_{13} + k_{14}[AtrbohF_P] - (k_{16} + k_{17}[AOX])[ROS], \tag{S12}$$

$$\frac{\mathrm{d}[AOX]}{\mathrm{d}t} = k_{18} + k_{19}[ROS] - k_{20}[AOX]. \tag{S13}$$

Figure S1B corresponds to this reduced model where some nodes and edges have been removed but the relationship between the signals and the output is still the same as in Fig. S1A. Though a simplification, this model is still large and we would like to find a further simplification. In the network representation of equations (S9)-(S13) shown in Fig. S1B the path-length from ABA to AtrbohF is 3 while the path-length from ethylene to AtrbohF is 1; however, in our experiments we observe a negligible difference between the ROS produced by ABA and the ROS produced by ethylene five minutes after treatment. We also note that the edge between ethylene and AtrbohF in Fig. S1B is positive because ETR1 inactivates the inactivator of AtrbohF in the absence of ethylene. These observations suggest that the number of steps between ethylene perception and ROS production is about the same as with ABA signals (the immediate events after ethylene binding by ETR1 in guard cells are still unknown), or if the number of events is different then the timescale of the reactions is approximately the same in both cases. Furthermore, in both cases the maximum rate of ROS production is limited by the total amount of AtrbohF ($[AtrbohF_T]$), which means that the response to either signal has the same theoretical maximum rate. Given that data are unavailable for the receptors, OST1, and ABI1, and that ABA and ethylene have similar timescales for producing ROS we further simplify our model so that AtrbohF becomes active directly from the ABA and ethylene signals, as shown in Fig. S1C.

To determine the equations that represent Fig. S1C, we must have a hypothesis of how ABA and ethylene signals activate AtrbohF. One possibility is that the signals activate AtrbohF through the same pathway, i.e., there is a bottleneck for both signals upstream of AtrbohF. In this case the signals are essentially interchangeable. This assumption would imply that, for example, a 2 μM dose of ABA and a combined 1 μM ABA plus 1 μM ethylene dose are the same:

$$\frac{\mathrm{d}[ROS]}{\mathrm{d}t} \approx k_{13} + \frac{[AtrbohF_T]([ABA + ACC])}{\kappa + ([ABA + ACC])} - (k_{16} + k_{17}[AOX])[ROS].$$

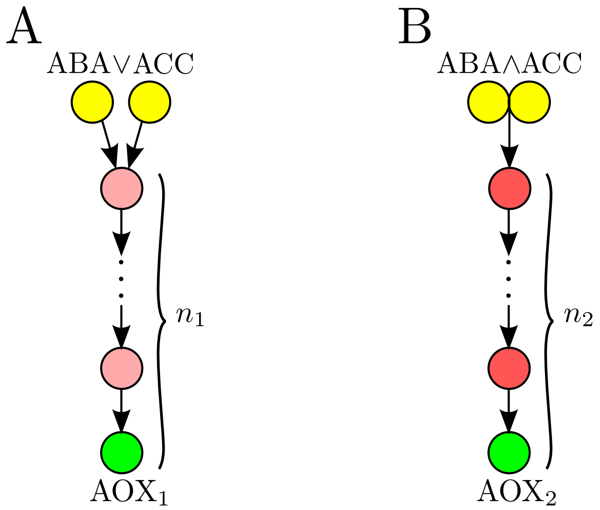

Figure S2: Antioxidant production at the end of linear activation cascades. **A**: Cascade of length n_1 whose input has a logic *or* function, the cascade responds to ABA or ethylene treatment (equation (S16)). **B**: Cascade of length n_2 whose input has a logic *and* function, the cascade becomes active only when ABA and ethylene are present simultaneously (equation (S17)).

The identity of the signalling "bottleneck" remains unknown. Another hypothesis that has a similar result but that does not require a common node in the pathways of the signals would be to assume that the signals converge for the first time at AtrbohF, and activate it independently of each other:

$$\frac{\mathrm{d}[ROS]}{\mathrm{d}t} = k_{13} + \frac{\alpha_1\kappa_2[ABA] + \alpha_2\kappa_1[ACC]}{\kappa_1\kappa_2 + \kappa_2[ABA] + \kappa_1[ACC]} - (k_{16} + k_{17}[AOX])[ROS], \tag{S14}$$

where α_1 is a product of $[AtrbohF_T]$ and other rate-limiting parameters in the ABA pathways, and κ_1 is the ABA-specific Michaelis constant, and likewise for ethylene α_2 is the rate-limiting parameter and κ_2 the Michaelis constant. We emphasise that this is not a rigorous derivation of the ROS-activation dynamics but a deduction guided by our current knowledge of the system and assumptions deemed reasonable. In Sec. 4 we derive the compound Michaelis-Menten term in equation (S14) using the QSSA (see Sec. 4). Though both hypotheses of ROS production can produce a similar response the latter one is better suited for modelling stomatal closure because it does not require the assumption of additional interactions, includes the former as a special case (when $\kappa_1 = \kappa_2$ and $\alpha_1 = \alpha_2 = [AtrbohF_T]$), and its derivation is more clear.

Now we turn our attention to the antioxidant pool AOX; it is clear that a homogeneous antioxidant pool responsive only to the concentration of ROS as described by equation (S13) is not compatible with our experimental observations. We consider the possibility of two different antioxidant mechanisms described by the variables $[AOX_1]$ and $[AOX_2]$ which lie at the end of linear activation cascades driven by $[ABA]$ and $[ACC]$ (Fig. S2). As discussed in the main text, there is evidence to suggest that two distinct antioxidant mechanisms might be at work during stomatal closure. The first of these mechanisms (AOX_1) would be activated by either an ABA *or* an ethylene signal and includes endogenous antioxidant production (to maintain unstimulated equilibrium levels), whereas the second antioxidant (AOX_2) is only active when ABA *and* ethylene signals are present simultaneously.

We place the response of the antioxidant to the signals at the end of cascades of abstract variables to emulate the delay observed in the removal of ROS. Each cascade has a constant input

(depending on the ABA and ethylene signals, see below) and has a solution proportional to the normalised lower-incomplete Gamma function $\mathrm{P}(n_i, h_i t)$ (see Sec. 3 where n_i represents the length of the cascade and h_i is related to the deactivation rates of the cascade, $i = 1, 2$.

The input of the cascade culminating in AOX_1 must follow a boolean *or* logic and saturate, therefore we use the compound Michaelis-Menten form we used to describe ROS-production previously as the input for the ABA and ethylene signals, so the equation for $[AOX_1]$ is

$$\frac{\mathrm{d}[AOX_1]}{\mathrm{d}t} = k_{18} + \frac{\alpha_3\kappa_4[ABA] + \alpha_4\kappa_3[ACC]}{\kappa_3\kappa_4 + \kappa_4[ABA] + \kappa_3[ACC]}\mathrm{P}(n_1, h_1 t) - k_{20}[AOX_1].$$

The cascade leading to the activation of AOX_2 must operate as a logic *and* gate, ie becoming active only if $[ABA] > 0$ and $[ACC] > 0$. We consider that the input of the cascade is downstream of the ABA and ethylene receptors and the response of the cascade must also exhibit saturation, so we model the input as the product of two Michaelis-Menten forms:

$$\left(\frac{\alpha_5[ABA]}{\kappa_5 + [ABA]}\right)\left(\frac{\alpha_6[ACC]}{\kappa_6 + [ACC]}\right),$$

which enforce a logical *and* operation of the signals. The expression for AOX_2 is

$$[AOX_2](t) = \frac{\alpha_5\alpha_6[ABA][ACC]}{(\kappa_5 + [ABA])(\kappa_6 + [ACC])}\mathrm{P}(n_2, h_2 t).$$

Now we have a complete reduced model of a ROS production-module in guard cells:

$$\frac{\mathrm{d}[ROS]}{\mathrm{d}t} = k_{13} + \frac{\alpha_1\kappa_2[ABA] + \alpha_2\kappa_1[ACC]}{\kappa_1\kappa_2 + \kappa_2[ABA] + \kappa_1[ACC]} - (k_{16} + k_{17}[AOX_1] + k_{21}[AOX_2])[ROS], \tag{S15}$$

$$\frac{\mathrm{d}[AOX_1]}{\mathrm{d}t} = k_{18} + \frac{\alpha_3\kappa_4[ABA] + \alpha_4\kappa_3[ACC]}{\kappa_3\kappa_4 + \kappa_4[ABA] + \kappa_3[ACC]}\mathrm{P}(n_1, h_1 t) - k_{20}[AOX_1], \tag{S16}$$

$$[AOX_2](t) = \frac{\alpha_5\alpha_6[ABA][ACC]}{(\kappa_5 + [ABA])(\kappa_6 + [ACC])}\mathrm{P}(n_2, h_2 t), \tag{S17}$$

with initial conditions $[ROS](0) = [ROS]_0$, $[AOX_1](0) = [AOX_1]_0$, and $[AOX_2](0) = 0$ still to be determined.

2.2 NO production

As discussed in the main text, ROS induces NO production in guard cells treated with ABA via the enzyme NIA1 (Fig. S3A); the possibility that NO is also produced in guard cells treated with ethylene was also discussed. An ODE describing endogenous and enzymatic NO production in guard cells dependent on ROS synthesis is

$$\frac{\mathrm{d}[NO]}{\mathrm{d}t} = \alpha_{30} + \frac{\alpha_{31}[ROS]}{k_{31} + [ROS]} - \beta_{30}[NO],$$

where α_{30} is a constant rate of NO production by other processes, the Michaelis-Menten term is ROS-induced NO production via NIA1, and the last term is NO decay and removal.

Note that in unstimulated guard cells $[ROS] > 0$ and as measurements of NO to distinguish between ROS and non-ROS induced production are not available, we gather the ROS-dependent and ROS-independent NO production in a single term. We include a second term that describes further enzymatic NO production from ethylene (Fig. S3B), which could be either from NIA1 or another, yet unidentified source (see main text). The new expression for NO production becomes

$$\frac{\mathrm{d}[NO]}{\mathrm{d}t} = \frac{\alpha_{31}[ROS]}{k_{31} + [ROS]} + \frac{\alpha_{32}[ACC]}{k_{32} + [ACC]} - \beta_{30}[NO], \tag{S18}$$

with initial condition $[NO](0) = [NO]_0$.

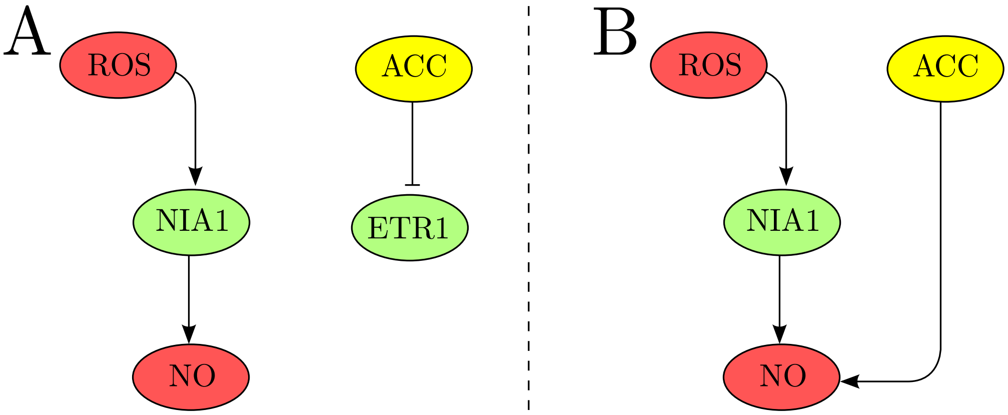

Figure S3: NO production models in guard cells. **A**: NO-production unit. NO is produced in a ROS-dependent way by NIA1. **B**: Model of NO-production given in equation (S18). The key assumption of this model is the existence of a ROS-independent pathway of NO production in response to ethylene.

2.3 Ca^{2+} increase, cytosolic alkalinisation, and ion efflux

The dynamics of Ca^{2+}-release and action in guard cells are complex and not yet fully understood [10]. Though the importance of Ca^{2+} in guard cell signalling (and cell viability in general) is beyond doubt, in this work we do not include an equation describing its behaviour for three reasons:

i. Reports of Ca^{2+}-behaviour after ABA treatments in the literature describe both oscillations and rises in cytosolic levels, and experimental data-sets encompassing both single and combined ABA and ethylene treatments do not exist.

ii. As mentioned in Ref. [10], the way in which a cytosolic Ca^{2+} rise (or oscillations) transmits signals during stomatal closure is not yet clear. Current hypotheses state that ABA "primes" receptors of Ca^{2+}, making the rise in cytosolic levels helpful but not essential for successful closure.

iii. Our experiments suggest that ABA-ethylene cross-talk occurs at the ROS-level, upstream of Ca^{2+} in the guard cell signal transduction network (Fig. 2 of the main text), so we direct most of our efforts to understanding signal transduction at this level.

During ABA-induced stomatal closure the pH in the cytosol of guard cells increases from 7.0 to 7.5, and the pH in the tonoplast decreases from 5.5 to 5.0 after treatment with ABA. The concentration of H^+ determines pH through the Henderson-Hasselbach equation: $pH = -\log([H^+])$ [19]. During stomatal closure the membrane H^+–ATPases are inactivated, which means that changes in pH are the result of the transport of protons from the cytosol into the tonoplast by the vacuolar proton pumps (V–ATPases), activated by OST1 [24]. The model of cytosolic alkalinisation and ion-efflux shown in Fig. S4B has one equation for the potassium ion concentration $[K^+]$, and one equation for the outwards potassium channels $[K^+_{out}]$:

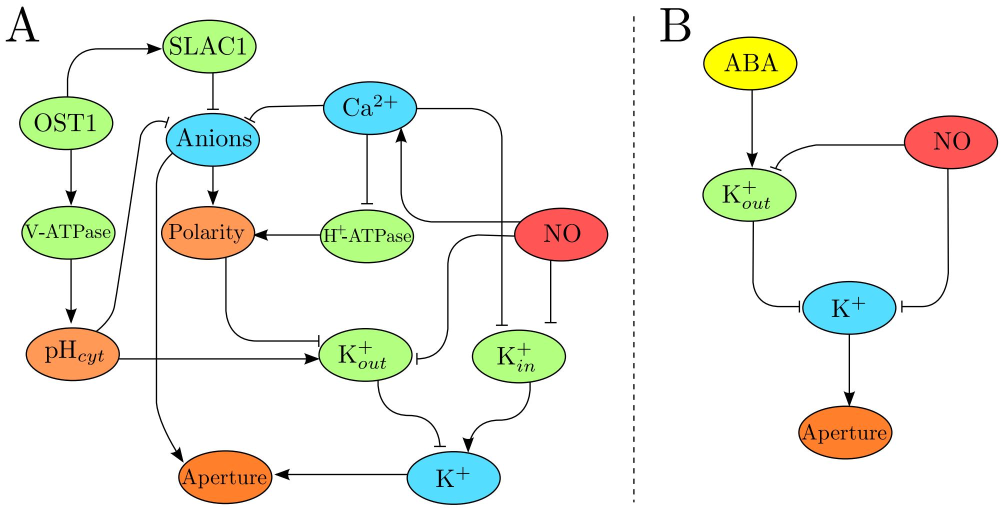

Figure S4: Events in stomatal closure downstream of ROS. **A**: Cytosolic alkalinisation following treatment with ABA, Ca^{2+}–increase, membrane depolarisation, and ion efflux. **B**: Model of the late events in stomatal closure presented in equations (S19) and (S20).

$$\frac{\mathrm{d}[K^+_{out}]}{\mathrm{d}t} = \alpha_{40} + \frac{\alpha_{41}[ABA]}{k_{11} + [ABA]} + \alpha_{42}[NO] - \beta_{40}[K^+_{out}], \tag{S19}$$

$$\frac{\mathrm{d}[K^+]}{\mathrm{d}t} = \frac{\alpha_{51}}{k_{51} + [NO]} - \beta_{50}[K^+_{out}][K^+]. \tag{S20}$$

Equation (S19) shows the change in $[K^+_{out}]$, the active outwards K^+ channels. The first and last terms represent the constant flux of channels between the active and inactive states, respectively. The second term represents the extra number of channels made available by the increase in cytosolic pH following an ABA stimulus, mediated by OST1. The third term ($\alpha_{42}[NO]$) is the increase in K^+_{out} activity as a result of membrane depolarisation, possibly via NO-induced Ca^{2+} release (ie via the path NO $\rightarrow$ Ca^{2+} $\dashv$ H^+–ATPase $\rightarrow$ Polarity $\dashv$ K^+_{out} in Fig. S4A). We assume that NO does not target K^+_{out}, as ion efflux is required for stomatal closure (we note that although NO has been shown to block K^+_{out} in *Vicia faba* guard cells [23], the authors of the study are unsure whether NO action is specifically targeted to K^+_{out}.) Equation (S20) shows the change in $[K^+]$. The first term represents the increase of ions that enter through the inwards-rectifying channels (K^+_{in}), which are inactivated by NO. The second term is the ion efflux through the outwards channels that is proportional to the active channels $[K^+_{out}]$ and the ion concentration itself. We include an equation for K^+_{out} but not for K^+_{in} because the alkalinisation of the cytosol has the effect of increasing the number of available channels to extrude ions, whereas the inactivation of K^+_{in} is only represented by a term in the equation for K^+.

2.4 Loss of turgor

The relationship of this model to stomatal aperture is via the last variable $[K^+]$. Cell volume (and hence stomatal aperture) is determined by the ion and solute concentration in the cell relative to the external concentration [16, 25]. Therefore, we take ions and solutes (K^+ in particular) as a simple proxy for aperture:

$$[AP] \propto [K^+]. \tag{S21}$$

We assume for this work that the relationship between ionic concentration and aperture is linear; as more data become available (K^+ and solute concentration) this relationship is likely to change in order to become more realistic, possibly becoming nonlinear.

2.5 A model of signal transduction for stomatal closure

We use equations (S15)-(S20) to construct the model of ABA and ethylene-induced stomatal closure represented graphically in Fig. 4 of the main text. We normalise the variables in the model by their non-stimulated equilibrium levels (ie the initial conditions) so they represent percentage of control:

$$\widehat{[ROS]}(t) = \frac{100[ROS](t)}{[ROS]_0}, \qquad \widehat{[AOX_1]}(t) = \frac{100[AOX_1](t)}{[AOX]_0},$$
$$\widehat{[NO]}(t) = \frac{100[NO](t)}{[NO]_0}, \qquad \widehat{[K^+_{out}]}(t) = \frac{100[K^+_{out}](t)}{[K^+_{out}]_0},$$
$$\widehat{[K^+]}(t) = \frac{100[K^+](t)}{[K^+]_0}, \qquad \widehat{[AP]}(t) = \frac{100[AP](t)}{[AP]_0}.$$

With the normalised variables we can transform equation (S21) to

$$\widehat{[K^+]}(t) = \widehat{[AP]}(t),$$

so we take the normalised potassium ion concentration as equivalent to the normalised aperture. Dropping the hat notation, the equations of the model become (note that the parameters have been renamed)

$$\frac{\mathrm{d}[ROS]}{\mathrm{d}t} = \alpha_{10} + \frac{\alpha_{11}k_{12}[ABA] + \alpha_{12}k_{11}[ACC]}{k_{11}k_{12} + k_{12}[ABA] + k_{11}[ACC]} - (\beta_{11}[AOX_1] + \beta_{12}[AOX_2])\,[ROS], \tag{S22}$$

$$\frac{\mathrm{d}[AOX_1]}{\mathrm{d}t} = \alpha_{20} + \frac{\alpha_{21}k_{22}[ABA] + \alpha_{22}k_{21}[ACC]}{k_{21}k_{22} + k_{22}[ABA] + k_{21}[ACC]}\,\mathrm{P}(n_1, \alpha_{23}t) - \beta_{20}[AOX_1], \tag{S23}$$

$$[AOX_2](t) = \frac{[ABA][ACC]}{(k_{11} + [ABA])(k_{12} + [ACC])}\mathrm{P}(n_2, \beta_{13}t). \tag{S24}$$

$$\frac{\mathrm{d}[NO]}{\mathrm{d}t} = \frac{\alpha_{31}[ROS]}{k_{31} + [ROS]} + \frac{\alpha_{32}[ACC]}{k_{12} + [ACC]} - \beta_{30}[NO], \tag{S25}$$

$$\frac{\mathrm{d}[K^+_{\mathrm{out}}]}{\mathrm{d}t} = \alpha_{40} + \frac{\alpha_{41}[ABA]}{k_{11} + [ABA]} + \alpha_{42}[NO] - \beta_{40}[K^+_{\mathrm{out}}], \tag{S26}$$

$$\frac{\mathrm{d}[K^+]}{\mathrm{d}t} = \frac{\alpha_{51}}{k_{51} + [NO]} - \beta_{50}[K^+_{\mathrm{out}}][K^+], \tag{S27}$$

which is the model we present in equations (1)-(6) of the main text.

2.6 Alternative hypothesis: Ethylene-induced cytosolic alkalinisation

An alternative version of the model in equations (S22)-(S27) that approximates the experimental data equally well includes ethylene-induced pH rise. The equations in the alternative model are the same with the exception that $\alpha_{32} = 0$ in equation (S25) and equation (S26) becomes:

$$\frac{\mathrm{d}[K^+_{\mathrm{out}}]}{\mathrm{d}t} = \alpha_{40} + \frac{\alpha_{41}[ABA]}{k_{11} + [ABA]} + \alpha_{42}[NO] + \frac{\alpha_{43}[ACC]}{k_{12} + [ACC]} - \beta_{40}[K^+_{\mathrm{out}}], \tag{S28}$$

which has the same number of parameters as the original one. Yet another variant would be to use equation (S28) and have $\alpha_{32} > 0$ in equation (S25) which fits the data equally well.

2.7 Allostery hypothesis for ABA-ethylene cross-talk in ROS production

We have explored an alternative hypothesis to explain the decrease in ROS production in guard cells under a combined ABA-ACC treatment. Motivated by the findings in Ref. [22] which show that the NADPH-oxidase *AtrbohF* has two phosphorylation sites, we explore the idea that ROS can only be produced when *AtrbohF* has a single phosphorylation, and that double phosphorylation returns the enzyme to a non ROS-producing state (Fig. S5A). In this scenario, an ABA signal can only lead to the phosphorylation of only one of the two active sites, and likewise an ethylene signal can only lead to the phosphorylation of the other active site only. When the ABA and ethylene signals are present simultaneously time then *AtrbohF* may become doubly-phosphorylated: once by ABA and once by ethylene, rendering it unable to produce ROS. We use a simple mass-action model to test this hypothesis:

$$\frac{\mathrm{d}[N_{PA}]}{\mathrm{d}t} = \alpha_{11}[ABA][N_T] - \beta_{11}[N_{PA}] - \beta_{12}[ACC][N_{PA}], \tag{S29}$$

$$\frac{\mathrm{d}[N_{PE}]}{\mathrm{d}t} = \alpha_{21}[ACC][N_T] - \beta_{21}[N_{PE}] - \beta_{22}[ABA][N_{PE}], \tag{S30}$$

$$\frac{\mathrm{d}[N_{PP}]}{\mathrm{d}t} = \beta_{12}[ACC][N_{PA}] + \beta_{22}[ABA][N_{PE}] - \beta_{31}[N_{PP}], \tag{S31}$$

$$\frac{\mathrm{d}[AOX]}{\mathrm{d}t} = \alpha_{50} + \frac{\alpha_{52}k_{52}[ABA] + \alpha_{53}k_{51}[ACC]}{k_{51}k_{52} + k_{52}[ABA] + k_{51}[ACC]}\mathrm{P}(\alpha_{54}t, n_5) - \beta_{50}[AOX], \tag{S32}$$

$$\frac{\mathrm{d}[ROS]}{\mathrm{d}t} = \alpha_{40} + \alpha_{41}[N_{PA}] + \alpha_{42}[N_{PE}] - (\beta_{41} + \beta_{42}[AOX])\,[ROS], \tag{S33}$$

where $[N_T]$ is the amount of total *AtrbohF*, $[N_{PA}]$; the concentration of *AtrbohF* with a single phosphorylation caused by ABA signals, $[N_{PE}]$; the concentration of *AtrbohF* with a single ethylene phosphorylation, and $[N_{PP}]$; the concentration of doubly-phosphorylated *AtrbohF*.

Figure S5B shows the time-courses of the model above to the ROS production along with data obtained from our experiments. The single stimulus solutions of the equations (to ABA or ACC stimuli alone) have a reasonable fit to the data but under a combined stimulus, the equations are not able to reproduce the peak in ROS production five minutes after treatment.

3 Modelling of activation through linear cascades

We model $[AOX_2]$ and the production of $[AOX_1]$ as the result of a linear activation cascade with a constant input λ. A model for such cascade is given by the following system of ODEs [9, 5]:

$$\begin{aligned}\frac{\mathrm{d}x_1}{\mathrm{d}t} &= \lambda - \beta_1 x_1,\\ \frac{\mathrm{d}x_2}{\mathrm{d}t} &= \alpha_2 x_1 - \beta_2 x_2,\\ &\vdots\\ \frac{\mathrm{d}x_n}{\mathrm{d}t} &= \alpha_n x_{n-1} - \beta_n x_n.\end{aligned} \tag{S34}$$

with initial conditions $x_i(0) = 0$, $\forall i$. If we assume that all the deactivation rates of all components in the cascade are identical (i.e., $\beta_i = \beta$, $\forall i$) then the cascade provides optimal amplification [5]

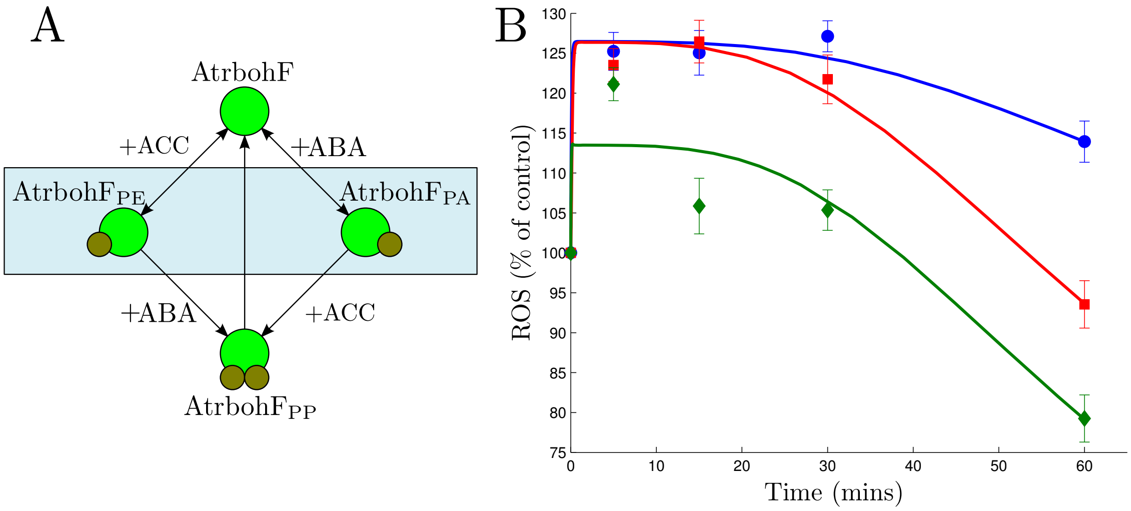

Figure S5: (Colour) **A**: Allostery hypothesis for ROS production. When *AtrbohF* has a single phosphorylation (in blue rectangle) it is capable of producing ROS, but under a combined treatment it can phosphorylate twice, becoming inactive. **B**: Time course of ROS production from equations (S29)-(S33) (solid lines) along with data under a 10μM stimuli of ABA (blue circles), ACC (red squares), and both (green diamonds).

and the exact solution for the last component ($x_n(t)$) is [2]:

$$x_n(t) = \frac{\lambda}{\beta^n}\left(\prod_{j=2}^{n}\alpha_j\right)\mathrm{P}(n,\beta t), \tag{S35}$$

where $\mathrm{P}(n,\beta t)$ is the normalised lower incomplete Gamma function [17].

For $[AOX_2]$, in Eq. (S7) the input signal is $\lambda = \frac{[ABA][ACC]}{(k_{11}+[ABA])(k_{12}+[ACC])}$ to give

$$[AOX_2](t) = \frac{\beta_{12}[ABA][ACC]}{(k_{11}+[ABA])(k_{12}+[ACC])}\mathrm{P}(n_2,\beta_{13}t)$$

where we condense the reactivity, activation and degradation rates in:

$$\beta_{12} = \frac{1}{\beta^{n_2}}\left(\prod_{j=2}^{n_2}\alpha_j\right).$$

with the timescale of the cascade $\beta_{13} = \beta$.

For the production term of $[AOX_1]$ in Eq. (S8), we have an input signal

$$\lambda = \frac{\frac{\alpha_{21}}{\rho}k_{22}[ABA] + \frac{\alpha_{22}}{\rho}k_{21}[ACC]}{k_{21}k_{22}+k_{22}[ABA]+k_{21}[ACC]},$$

with $\rho > 0$ such that

$$\rho = \frac{1}{\beta^{n_1}}\left(\prod_{j=2}^{n_1}\alpha_j\right),$$

the timescale $\alpha_{23} = \beta$. From which we obtain the term in the equation:

$$\frac{\alpha_{21}k_{22}[ABA] + \alpha_{22}k_{21}[ACC]}{k_{21}k_{22}+k_{22}[ABA]+k_{21}[ACC]}\mathrm{P}(n_1,\alpha_{23}t).$$

4 Compound Michaelis-Menten forms

In this section we derive the compound Michaelis-Menten term we use in our model of ABA and ethylene-induced stomatal closure. Suppose an enzyme E catalyses the production of a substance P from two different substrates S_1 and S_2 in the following chemical reactions:

$$S_1 + E \underset{k_2}{\overset{k_1}{\rightleftharpoons}} C_1 \overset{k_3}{\longrightarrow} P + E$$
$$S_2 + E \underset{k_5}{\overset{k_4}{\rightleftharpoons}} C_2 \overset{k_6}{\longrightarrow} P + E.$$

The enzyme binds to the substrates to form a complex which can either dissociate or catalyse the reaction. If $[E]$, $[S_1]$, $[S_2]$, $[C_1]$, $[C_2]$, and $[P]$ denote the concentrations of the reactants and the products of the reactions, and the substrates are abundant enough (ie the concentrations can be considered to remain constant), then the ODE system describing the reactions is:

$$\frac{\mathrm{d}[E]}{\mathrm{d}t} = -k_1[S_1][E] - k_4[S_2][E] + (k_2 + k_3)[C_1] + (k_5 + k_6)[C_2], \quad \text{(S36)}$$
$$\frac{\mathrm{d}[C_1]}{\mathrm{d}t} = k_1[S_1][E] - (k_2 + k_3)[C_1], \quad \text{(S37)}$$
$$\frac{\mathrm{d}[C_2]}{\mathrm{d}t} = k_4[S_2][E] - (k_5 + k_6)[C_2], \quad \text{(S38)}$$
$$\frac{\mathrm{d}[P]}{\mathrm{d}t} = k_3[C_1] + k_6[C_2]. \quad \text{(S39)}$$

Because $\frac{\mathrm{d}[E]}{\mathrm{d}t} + \frac{\mathrm{d}[C_1]}{\mathrm{d}t} + \frac{\mathrm{d}[C_2]}{\mathrm{d}t} = 0$, we express the total concentration of enzyme as $E_T = [E] + [C_1] + [C_2]$, $E_T \in \mathbb{R}^+$. We eliminate equation (S36) using this conservation relation. Now equations (S37) and (S38) become:

$$\frac{\mathrm{d}[C_1]}{\mathrm{d}t} = k_1[S_1](E_T - [C_1] - [C_2]) - (k_2 + k_3)[C_1], \quad \text{(S40)}$$
$$\frac{\mathrm{d}[C_2]}{\mathrm{d}t} = k_4[S_2](E_T - [C_1] - [C_2]) - (k_5 + k_6)[C_2]. \quad \text{(S41)}$$

The quasi-steady-state approximation (QSSA) states that before any meaningful amounts of P are produced, the enzymes and complexes reach an equilibrium [14, 21]. Thus, equations (S40) and (S41) are both equal to zero, because they do not depend on equation (S39) and we can analyse them in isolation:

$$0 = k_1[S_1](E_T - [C_1] - [C_2]) - (k_2 + k_3)[C_1],$$
$$0 = k_4[S_2](E_T - [C_1] - [C_2]) - (k_5 + k_6)[C_2].$$

If $k_a = (k_2 + k_3)/k_1$ and $k_b = (k_5 + k_6)/k_4$ then

$$[C_1] = \frac{k_b E_T [S_1]}{k_b[S_1] + k_a[S_2] + k_a k_b}, \quad \text{(S42)}$$
$$[C_2] = \frac{k_a E_T [S_2]}{k_b[S_1] + k_a[S_2] + k_a k_b}. \quad \text{(S43)}$$

The production of P can be expressed as

$$\frac{\mathrm{d}[P]}{\mathrm{d}t} \approx \frac{E_T(k_3 k_b[S_1] + k_6 k_a[S_2])}{k_b[S_1] + k_a[S_2] + k_a k_b}, \quad \text{(S44)}$$

which is a compound Michaelis-Menten form. When $[S_1] > 0$ and $[S_2] = 0$ the expression in equation (S42) becomes proportional to the standard Michaelis-Menten form:

$$[C_1] = \frac{E_T k_3 [S_1]}{[S_1] + k_a},$$

likewise when $[S_1] = 0$ and $[S_2] > 0$ we have

$$[C_2] = \frac{E_T k_6 [S_2]}{[S_2] + k_b}.$$

5 Parameter fitting

Equations (1)–(6) of the main text have 28 parameters whose values must be determined. (Note that the variables are rescaled dividing them by 100, so that control levels and initial conditions are 1, to improve numerical stability of the fitting process.)

We use our experimental time-course measurements of ROS and aperture to fit the parameters in Eqs. (1)–(6) of the main text. Our numerical evaluations of the model always start from a 'control' initial condition:

$$[ROS](0) = [AOX_1](0) = [NO](0) = [K^+_{out}](0) = [K^+](0) = 100, \text{ and } [AOX_2](0) = 0, \tag{S45}$$

and we introduce constraints to guarantee that the system remains unchanged at the control initial condition when there is no treatment (i.e., $[ABA] = [ACC] = 0$):

$$\frac{\mathrm{d}[ROS]}{\mathrm{d}t} = \frac{\mathrm{d}[AOX]}{\mathrm{d}t} = \frac{\mathrm{d}[NO]}{\mathrm{d}t} = \frac{\mathrm{d}[K^+_{out}]}{\mathrm{d}t} = \frac{\mathrm{d}[K^+]}{\mathrm{d}t} = 0, \tag{S46}$$

Hence the following relationships between parameters must hold:

$$\begin{aligned} \alpha_{10} &= \beta_{11}, \\ \alpha_{20} &= \beta_{20}, \\ \beta_{30} &= \frac{\alpha_{31}}{k_{31}+1}, \\ \beta_{40} &= \alpha_{40} + \alpha_{42}, \\ \beta_{50} &= \frac{\alpha_{51}}{k_{51}+1}. \end{aligned} \tag{S47}$$

With these conditions, the number of unknown parameters has been reduced from 28 to 23 and we define the vector $\boldsymbol{\theta} \in \mathbb{R}^{23}$ in parameter space:

$$\boldsymbol{\theta} = [\alpha_{11}, \alpha_{12}, k_{11}, k_{12}, \beta_{11}, \beta_{12}, \beta_{13}, n_2, \alpha_{22}, \alpha_{23}, k_{21}, k_{22}, \alpha_{24}, n_1, \beta_{20}, \alpha_{31}, k_{31}, \alpha_{32}, \alpha_{41}, \alpha_{42}, \alpha_{43}, \alpha_{51}, k_{51}],$$

whose components are all non-negative. We define the set of treatments as $\mathcal{T} = \{T_1, T_2, T_3, T_4, T_5\}$ where:

$$\begin{aligned} T_1 &= 1\,\mu M \text{ ABA} + 0\,\mu M \text{ ACC}, \\ T_2 &= 0\,\mu M \text{ ABA} + 1\,\mu M \text{ ACC}, \\ T_3 &= 10\,\mu M \text{ ABA} + 0\,\mu M \text{ ACC}, \\ T_4 &= 0\,\mu M \text{ ABA} + 10\,\mu M \text{ ACC}, \\ T_5 &= 10\,\mu M \text{ ABA} + 10\,\mu M \text{ ACC}. \end{aligned}$$

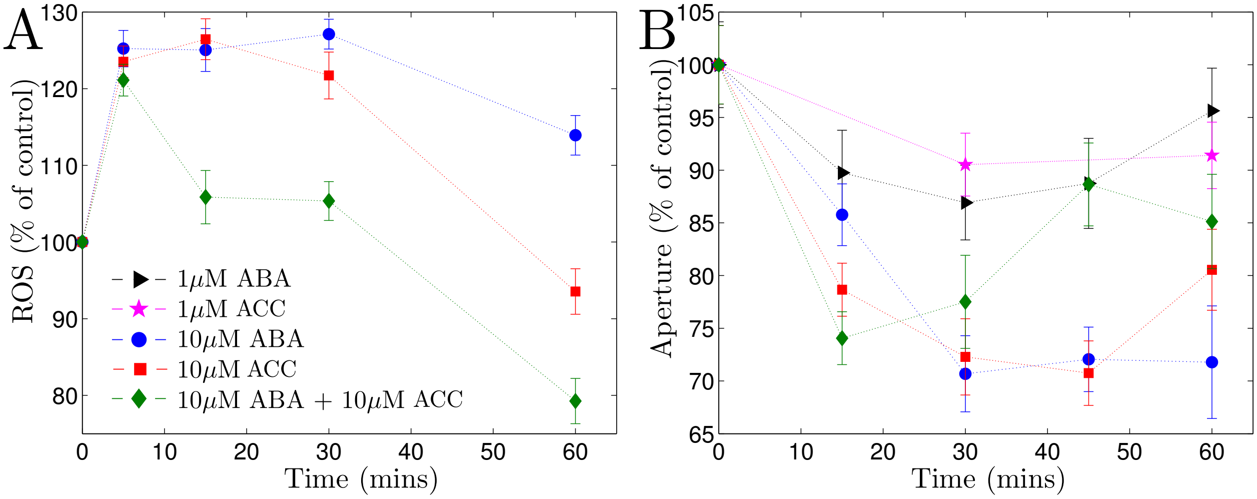

Figure S6: Colour **A**: Time-course of ROS fluorescence as percent of control. Blue circles show measurements made after a 10μM ABA stimulus, red squares; after a 10μM ACC stimulus, and green diamonds; after a combined 10μM ABA + 10μM ACC stimulus. **B**: Time-course of stomatal aperture after the three doses used in **A** plus 1μM ABA (black triangles), and 1μM ACC (pink stars). In all measurements error bars denote the standard error of the mean ($n = 30 \times 3$).

The data set $\mathcal{D}$ with measurements of ROS and aperture consists of the observations shown in Fig. S6.

We denote by $[\widehat{ROS}]_i$ and $[\widehat{AP}]_i$ the vectors of measurements of ROS and aperture under treatment T_i; and by $[ROS]_i(\boldsymbol{\theta})$ and $[AP]_i(\boldsymbol{\theta})$ the model predictions at the same time points as the data with dose T_i, using parameters $\boldsymbol{\theta}$. The discrepancy of the model in equations (1)–(6) of the main text and the data is measured by the following error function:

$$E_{\mathcal{D}}(\boldsymbol{\theta}) = \sum_{T_i} \left|\left|[\widehat{ROS}]_i - [ROS]_i(\boldsymbol{\theta})\right|\right|_2^2 + \left|\left|[\widehat{AP}]_i - [AP]_i(\boldsymbol{\theta})\right|\right|_2^2, \tag{S48}$$

where $||\cdot||_2$ is the euclidean norm. That is, we measure the distance between our ROS and aperture measurements and the model for a given $\boldsymbol{\theta}$ in the parameter space. The global optimisation problem is to find $\boldsymbol{\theta}^{\ddagger}$ where

$$\boldsymbol{\theta}^{**} = \min_{\boldsymbol{\theta}} E_{\mathcal{D}}(\boldsymbol{\theta}),$$
$$\text{subject to } \boldsymbol{\theta} \geq 0.$$

We use the Squeeze-and-breathe optimisation method [3] to find $\boldsymbol{\theta}^{\ddagger}$. The method requires an initial probability distribution for each parameter (called a *prior*). In this work, we used a uniform distribution $U(0, 10)$ for all parameters. On each iteration 500 points in the parameter space (in $\mathbb{R}^{23}_{+}$) are sampled from the prior. Each point is used as a starting point to minimise $E_{\mathcal{D}}(\boldsymbol{\theta})$ locally (using the Nelder-Mead simplex algorithm). The 50 local minima with the smallest errors are used to construct a *posterior* distribution of the parameters. The posterior is used as a prior for the next iteration where another 500 points are sampled and minimised until the convergence criteria has been met. Figure S7 shows the convergence of the method for fitting the parameters of our model. On Fig. S7A, we show the decrease in the difference (on a semilogarithmic scale) between the errors of the parameter sets found at the end of each iteration and the global minimum $E_{\mathcal{D}}(\boldsymbol{\theta}^{\ddagger}) \approx 0.0215$,

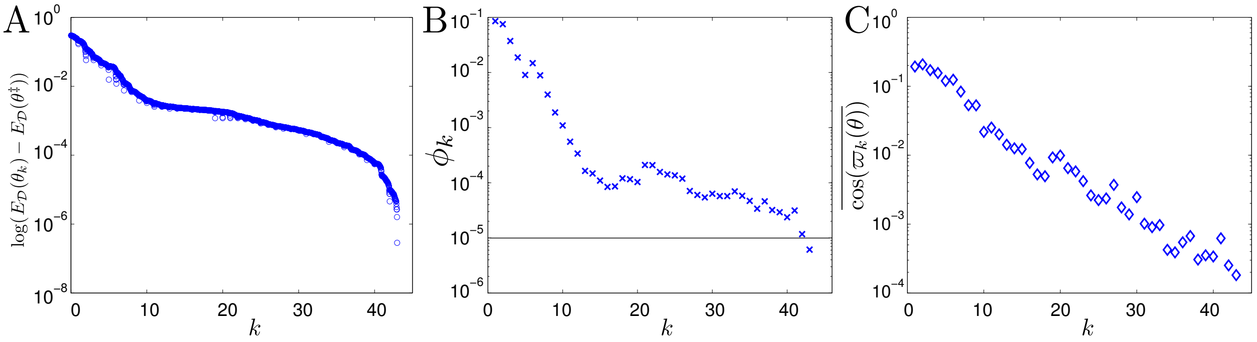

Figure S7: Convergence criteria for optimisation algorithm. **A**: Difference between the best 50 local minima from each iteration (k) of the algorithm (the posterior) and the global minimum $\boldsymbol{\theta}^{\ddagger}$ on a semilogarithmic scale. **B**: Difference between the mean of the errors of the local minima from consecutive iterations of the fitting algorithm (ϕ_k) on a semilogarithmic scale. **C**: Mean of the cosines among individual local minima from each iteration on a semilogarithmic scale.

obtained at the end of iteration 43. The 50 lowest errors of each iteration minus $E_{\mathcal{D}}(\boldsymbol{\theta}^{\ddagger})$ are shown on a decreasing order from left to right. Figure S7B shows the convergence criterion defined in Ref. [3]. We stop the iterations of the method once the difference between mean of the errors of the 50 parameter sets from consecutive iterations (ϕ_k, shown on a semilogarithmic scale) is smaller that 10^{-5}. During the first 20 iterations of the method ϕ_k appears to decrease exponentially and thereafter the trend still continues downwards albeit no longer exponentially. On Fig. S7C we show the mean of the cosines of the angles between all local minima from each iteration. This is to assert that the method converges to a single region of the parameter space. After iteration 43 the mean cosine is $O(10^{-4})$. Based on these metrics we conclude that $\boldsymbol{\theta}^{\ddagger}$ is a good estimation of the model parameters, given the present data. See Ref. [3] for a detailed description of the method.

Figure S8 shows the distribution of the best 50 parameters after 43 iterations of the algorithm. Red dots mark the mean of each parameter (values in Table 1). The behaviour of the model that we observe in Figs. 3, 5, and 6 of the main text is given by these parameters.

6 Model selection

We use Akaike's information criterion with a correction for finite sample sizes (AICc) to select the best out of the models presented here. The quantity measured by the AICc balances how well a model fits the data with the number of parameters to discourage overfitting and is a convenient way to perform model selection [4]. The AICc of a model i is given by:

$$AICc_i = n\ln(nRSS_i) + 2p_i + \frac{2p_i(p_i+1)}{n-(p_i+1)},$$

where n is the number of observations, p_i is the number of parameters in model i, and RSS_i is the residual sum of squares of the model. Given many models, the one with the lowest AICc$_i$ is considered preferable [4].

We computed the AICc for several versions of the models considered in order to obtain equations (S22)-(S27) and (S28) (which were constructed methodically from known interactions), under different assumptions concerning the value of some of the parameters which reflect different hypotheses about the way in which ABA and ethylene signals are processed in stomata (all values are summarised in Table S2):

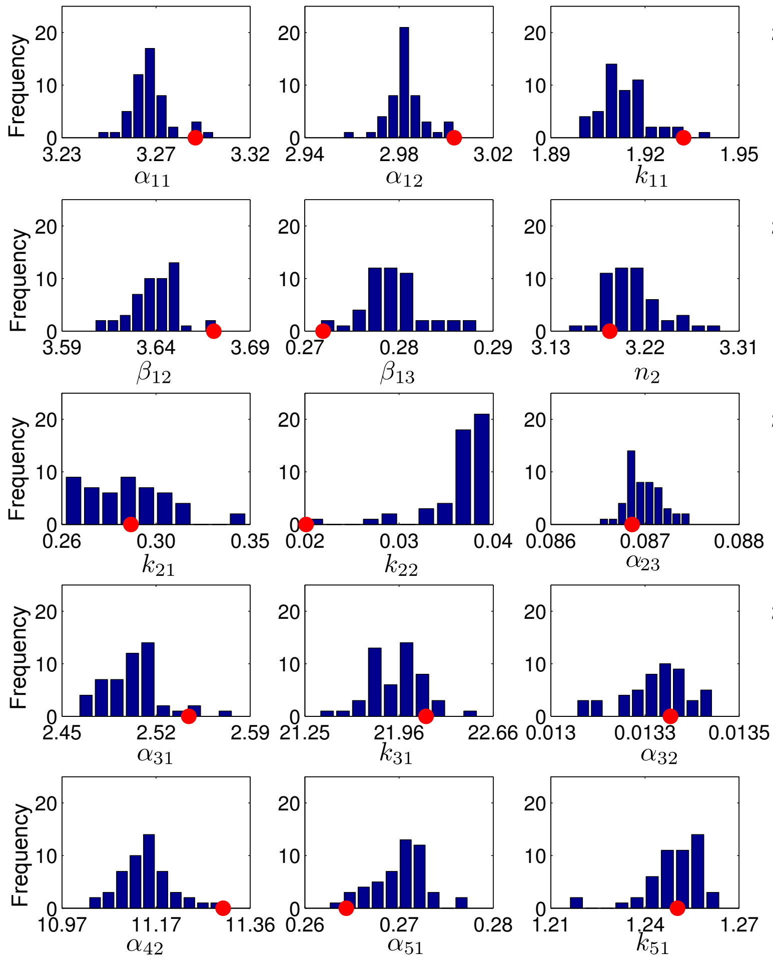

Figure S8: (Colour) Histograms of the distribution of the 50 best parameters obtained after 43 iterations of the fitting algorithm. The red dots indicate the value of the parameter that gave the smallest error ($\boldsymbol{\theta}^{\ddagger}$).

Parameter	Value	Units
α_{11}	3.2976	min^{-1}
α_{12}	3.0054	min^{-1}
k_{11}	1.9389	dimensionless
k_{12}	1.551	dimensionless
β_{11}	10.8119	min^{-1}
β_{12}	3.6752	min^{-1}
β_{13}	0.2749	dimensionless
n_2	3.192	dimensionless
α_{21}	3.8179	min^{-1}
α_{22}	11.9759	dimensionless
k_{21}	0.2962	dimensionless
k_{22}	0.0268	dimensionless
α_{23}	0.0868	dimensionless
n_1	9.755	dimensionless
β_{20}	1.5973	min^{-1}
α_{31}	2.5528	min^{-1}
k_{31}	22.1635	dimensionless
α_{32}	0.0133	min^{-1}
α_{40}	5.7029	min^{-1}
α_{41}	1.4584	min^{-1}
α_{42}	11.3133	min^{-1}
α_{51}	0.2713	min^{-1}
k_{51}	1.256	dimensionless
α_{10}	10.8119	min^{-1}
α_{20}	1.5973	min^{-1}
β_{30}	0.1102	min^{-1}
β_{40}	17.0162	min^{-1}
β_{50}	0.1203	min^{-1}

Table S1: Parameter values obtained by the Squeeze-and-Breathe algorithm. Other values are given by the relations given in the Methods section. The bottom five parameters are calculated using the expressions in (S47).

Model	Number of parameters	AICc
9	21	268.6
10	21	142.4
11	21	196.0
12	22	158.6
13	23	138.8
14	20	164.4
15	23	121.2

Table S2: Number of parameters and values of Akaike's Information Criterion with correction for small sample size (AICc) of the 7 models described in Sec. 6. The number of data points in all the models is $n = 36$.

- Model 9: 21 parameters, $AICc_9 = 268.6$. No saturation in AOX_2 activation, and fixed cascade length of 2, no saturation in ethylene-induced production of NO, and no NO effect on K^+ channels.
- Model 10: 21 parameters, $AICc_{10} = 142.3$. Same as Model 9 except that we assume that the events that activate AOX_1 after ABA or ethylene stimuli have the same speed (equal Michaelis constants), and NO has an effect on K^+.
- Model 11: 21 parameters, $AICc_{11} = 196.0$. Model with ethylene-induced NO production, and no saturation (mass-action) in ABA-induced pH increase.
- Model 12: 22 parameters, $AICc_{12} = 158.6$. Length of cascade of AOX_2 is a parameter (n_2) , ABA-induced pH rise follows Michaelis-Menten kinetics, and the Michaelis constant in ethylene-induced NO is k_{12}, the same as in the ethylene receptors.
- Model 13: 23 parameters, $AICc_{13} = 138.8$. This is the model in equations (S22)-(S27).
- Model 14: 20 parameters, $AICc_{14} = 163.4$. Same as Model 13, but with $\alpha_{21} = \alpha_{11}$, $\alpha_{22} = \alpha_{12}$, $k_{21} = k_{11}$, $k_{22} = k_{12}$ (ie identical pathway between signal perception and ROS and AOX_1 production).
- Model 15: 23 parameters, 121.2. This is the alternative hypothesis discussed in Sec. 2.6.

Based on the AICc, the best models are 15, 13 and 10. Model 10 is discarded because it exhibits stomatal reopening with increased doses of ABA and ethylene, something that has been disproved experimentally. The error of models 13 and 15 are so close that although $AICc_{15} < AICc_{13}$, we consider them equally plausible until further experiments shed light into the behaviour of NO and K^+.

7 Sensitivity analysis

We perform a sensitivity analysis of the model in equations (S22)-(S27) (models 13 and 15 from Sec. 6) using the extended Fourier amplitude sensitivity test (eFAST) [13, 26] computed in MATLAB with the Systems Biology Toolbox [20]. The eFAST method computes sensitivity scores between 0 and 1 based on how fast the Fourier decomposition frequency of each parameter propagates from variations in inputs [13].

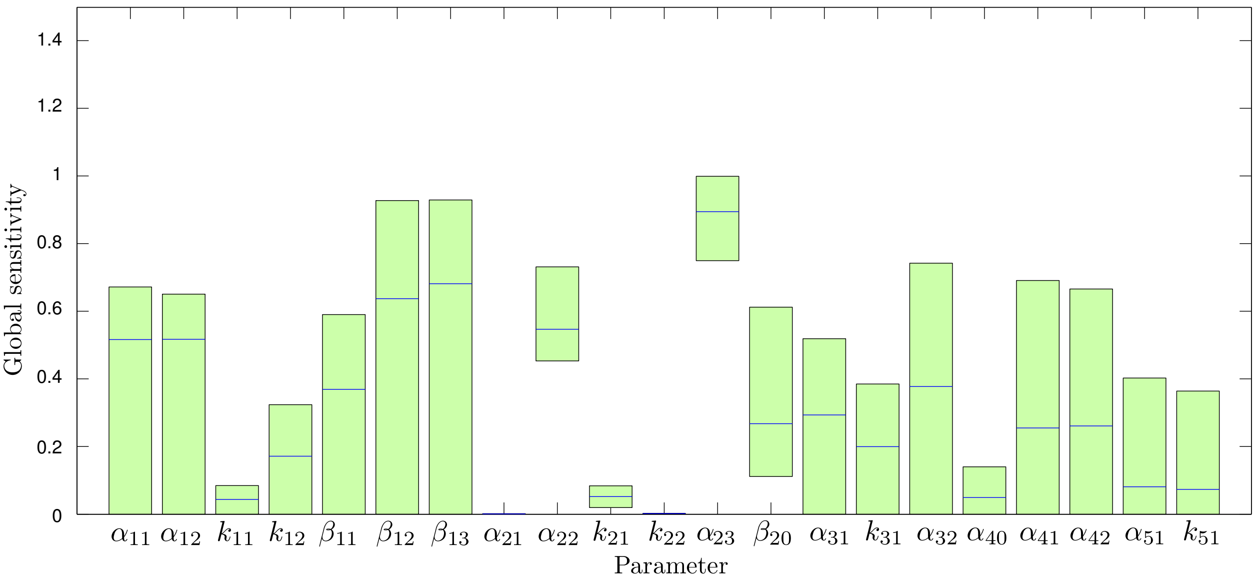

Figure S9: (Colour) Global sensitivities of the parameters in model (S22)-(S27) computed using the extended Fourier amplitude sensitivity test (eFAST). For each parameter, the mean sensitivity is given by the blue line, the bars cover the range between the minimum and the maximum sensitivity observed (1000 simulations, see Refs. [20, 13]). The parameter sensitivities in the model are low with the exception of those related to the antioxidant cascades (β_{12}, β_{13}, α_{23}) which, as described in the main text, are crucial to the observed behaviour of guard cells.

In Fig. S9 we show the sensitivities of the parameters in model (S22)-(S27) with fixed $n_1 = 9$ and $n_2 = 3$. The most sensitive parameter in the model is α_{23} (mean sensitivity 0.89), the deactivation rate in the cascade that produces AOX_1 in response to either ABA or ethylene. The next two most sensitive parameters are β_{12} and β_{13} (mean scores 0.63 and 0.68, respectively) which are the scavenging rate of ROS by AOX_2, and the degradation of the cascade components leading to the activation of AOX_2. Other sensitive parameters are related to ethylene-induced NO production and its effect on potassium ion extrusion.

In Fig. S10 we examine the sensitivities of the parameters in the model with the alternative hypothesis of pH rise induced by ethylene, discussed in Sec. 2.6. As in the main model, the most sensitive parameters are α_{23} and β_{13} (mean scores 0.71 and 0.59), related with the timescales of both antioxidants (the sensitivity of β_{12} is slightly less than in the previous model). The parameter α_{42} in equation (S28) which relates NO to K^+ extrusion shows increased variability in its sensitivity range. The parameter α_{43}, which is involved with the hypothetical effect of ethylene on pH (and thus on the number of K^+_{out} channels available) also appears to be sensitive.

References

[1] M. D. Abramoff, P. J. Magelhaes, and S. J. Ram, *Image Processing with ImageJ*, Biophotonics International, 11 (2004), pp. 36–42.

[2] M. Beguerisse-Díaz, P. J. Ingram, R. Desikan, and M. Barahona, *Linear models of activation cascades: analytical solutions and applications*, arXiv:1112.0270, (2011).

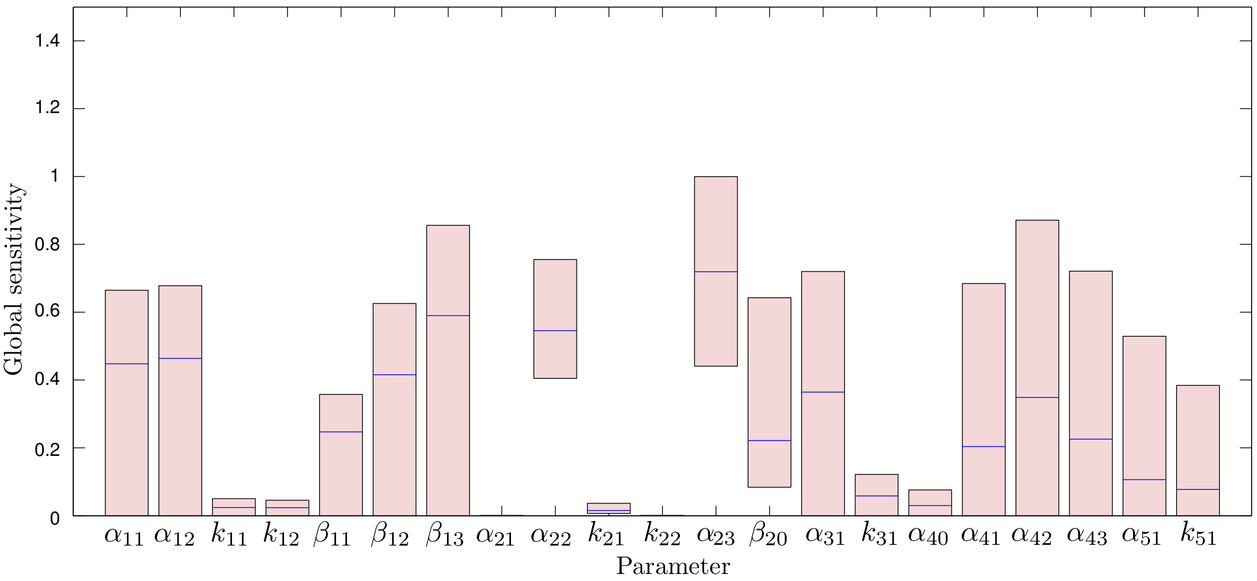

Figure S10: (Colour) Global sensitivities of the parameters in the model with the alternative hypothesis of ethylene-induced cytosolic alkalinisation from Sec. 2.6, computed using the extended Fourier amplitude sensitivity test (eFAST). The mean sensitivities (after 1000 simulations) are marked by the blue lines, the minimum and maximum observed sensitivities are covered by the bars. Here, as in the analysis in Fig. S9, the most sensitive parameters are those related to the antioxidants in the model, the other parameter's sensitivity remain relatively low.

[3] M. Beguerisse-Díaz, B. Wang, R. Desikan, and M. Barahona, *Squeeze-and-breathe evolutionary monte carlo optimization with local search acceleration and its application to parameter fitting*, Journal of The Royal Society Interface, 9 (2012), pp. 1925–1933.

[4] K. Burnham and D. Anderson, *Model Selection and Multi-Model Inference: A Practical Information-Theoretic Approach*, Springer, 2002.

[5] M. Chaves, E. D. Sontag, and R. J. Dinerstein, *Optimal Length and Signal Amplification in Weakly Activated Signal Transduction Cascades*, The Journal of Physical Chemistry B, 108 (2004), pp. 15311–15320.

[6] Y.-F. Chen, N. Etheridge, and G. E. Schaller, *Ethylene Signal Transduction*, Ann Bot, 95 (2005), pp. 901–915.

[7] R. Desikan, J. T. Hancock, J. Bright, J. Harrison, I. Weir, R. Hooley, and S. J. Neill, *A Role for ETR1 in Hydrogen Peroxide Signaling in Stomatal Guard Cells*, Plant Physiology, 137 (2005), pp. 831–834.

[8] R. Desikan, K. Last, R. Harrett-Williams, C. Tagliavia, K. Harter, R. Hooley, J. T. Hancock, and S. J. Neill, *Ethylene-induced stomatal closure in Arabidopsis occurs via AtrbohF-mediated hydrogen peroxide synthesis*, The Plant Journal, 47 (2006), pp. 907–916.

[9] R. Heinrich, B. G. Neel, and T. A. Rapoport, *Mathematical Models of Protein Kinase Signal Transduction*, Molecular Cell, 9 (2002), pp. 957–970.

[10] T.-H. Kim, M. Bhmer, H. Hu, N. Nishimura, and J. I. Schroeder, *Guard cell signal transduction network: advances in understanding abscisic acid, CO2, and Ca2+ signaling.*, Annual Review of Plant Biology, 61 (2010), pp. 561–591.

[11] J. M. Kwak, I. C.Mori, Z.-M. Pei, N. Leonhardt, M. A. Torres, J. L.Dangl, R. E.Bloom, S. Bodde, J. D.G.Jones, and J. I.Schroeder, *NADPH oxidase AtrbohD and AtrbohF genes function in ROS-dependent ABA signaling in Arabidopsis*, The EMBO Journal, 22 (2003), pp. 2623–2633.

[12] Y. Ma, I. Szostkiewicz, A. Korte, D. Moes, Y. Yang, A. Christmann, and E. Grill, *Regulators of PP2C Phosphatase Activity Function as Abscisic Acid Sensors*, Science, 324 (2009), pp. 1064–1068.

[13] S. Marino, I. B. Hogue, C. J. Ray, and D. E. Kirschner, *A Methodology for Performing Global Uncertainty and Sensitivity Analysis in Systems Biology*, Journal of Theoretical Biology, 254 (2008), pp. 178 – 196.

[14] J. Murray, *Mathematical Biology: I. An Introduction*, Springer, 2005.

[15] A.-C. Mustilli, S. Merlot, A. Vavasseur, F. Fenzi, and J. Giraudat, *Arabidopsis OST1 Protein Kinase Mediates the Regulation of Stomatal Aperture by Abscisic Acid and Acts Upstream of Reactive Oxygen Species Production*, Plant Cell, 14 (2002), pp. 3089–3099.

[16] S. Pandey, W. Zhang, and S. M. Assmann, *Roles of ion channels and transporters in guard cell signal transduction.*, FEBS Lett, 581 (2007), pp. 2325–2336.

[17] R. B. Paris, *Incomplete Gamma and Related Functions*, in NIST Digital Library of Mathematical Functions, 2010.

[18] S.-Y. Park, P. Fung, N. Nishimura, D. R. Jensen, H. Fujii, Y. Zhao, S. Lumba, J. Santiago, A. Rodrigues, T.-f. F. Chow, S. E. Alfred, D. Bonetta, R. Finkelstein, N. J. Provart, D. Desveaux, P. L. Rodriguez, P. McCourt, J.-K. Zhu, J. I. Schroeder, B. F. Volkman, and S. R. Cutler, *Abscisic Acid Inhibits Type 2C Protein Phosphatases via the PYR/PYL Family of START Proteins*, Science, 324 (2009), pp. 1068–1071.

[19] H. N. Po and N. M. Senozan, *The Henderson-Hasselbalch Equation: Its History and Limitations*, Journal of Chemical Education, 78 (2001), pp. 1499–.

[20] H. Schmidt and M. Jirstrand, *Systems Biology Toolbox for MATLAB: a computational platform for research in systems biology*, Bioinformatics, 22 (2006), pp. 514–515. http://www.sbtoolbox2.org.

[21] L. A. Segel and M. Slemrod, *The Quasi-Steady-State Assumption: A Case Study in Perturbation*, SIAM Review, 31 (1989), pp. 446–477.

[22] C. Sirichandra, D. Gu, H.-C. Hu, M. Davanture, S. Lee, M. Djaoui, B. Valot, M. Zivy, J. Leung, S. Merlot, and J. M. Kwak, *Phosphorylation of the Arabidopsis AtrbohF NADPH oxidase by OST1 protein kinase*, FEBS Lett, 583 (2009), pp. 2982–2986.

[23] S. Sokolovski and M. R. Blatt, *Nitric Oxide Block of Outward-Rectifying K+ Channels Indicates Direct Control by Protein Nitrosylation in Guard Cells*, Plant Physiology, 136 (2004), pp. 4275–4284.

[24] D. Suhita, A. S. Raghavendra, J. M. Kwak, and A. Vavasseur, *Cytoplasmic Alkalization Precedes Reactive Oxygen Species Production during Methyl Jasmonate- and Abscisic Acid-Induced Stomatal Closure*, Plant Physiology, 134 (2004), pp. 1536–1545.

[25] Y. Tanaka, T. Sano, M. Tamaoki, N. Nakajima, N. Kondo, and S. Hasezawa, *Cytokinin and auxin inhibit abscisic acid-induced stomatal closure by enhancing ethylene production in Arabidopsis*, Journal of Experimental Botany, 57 (2006), pp. 2259–2266.

[26] Y. Zheng and A. Rundell, *Comparative study of parameter sensitivity analyses of the tcr-activated erk-mapk signalling pathway*, Systems Biology, IEE Proceedings, 153 (2006), pp. 201 –211.
